# Genomic Characteristics and Potential Metabolic Adaptations of Hadal Trench *Roseobacter* and *Alteromonas* Bacteria Based on Single-Cell Genomics Analyses

**DOI:** 10.3389/fmicb.2020.01739

**Published:** 2020-07-24

**Authors:** Mingming Chen, Yu Song, Xiaoyuan Feng, Kai Tang, Nianzhi Jiao, Jiwei Tian, Yao Zhang

**Affiliations:** ^1^State Key Laboratory of Marine Environmental Science, College of Ocean and Earth Science, Xiamen University, Xiamen, China; ^2^School of Life Sciences, The Chinese University of Hong Kong, Hong Kong, China; ^3^Physical Oceanography Laboratory, Ocean University of China, Qingdao, China

**Keywords:** single-cell genome, *Roseobacter*, *Alteromonas*, metabolic adaptation, hadal trench

## Abstract

Heterotrophic bacteria such as those from the *Roseobacter* group and genus *Alteromonas* dominate the hadal zones of oceans; however, we know little about the genomic characteristics and potential metabolic adaptations of hadal trench-dwelling bacteria. Here, we report multiple single amplified genomes (SAGs) belonging to *Roseobacter* and *Alteromonas*, recovered from the hadal zone of the Mariana Trench. While phylogenetic analyses show that these hadal SAGs cluster with their surface relatives, an analysis of genomic recruitment indicates that they have higher relative abundances in the hadal zone of the Mariana Trench. Comparative genomic analyses between the hadal SAGs and reference genomes of closely related shallow-water relatives indicate that genes involved in the mobilome (prophages and transposons) are overrepresented among the unique genes of the hadal *Roseobacter* and *Alteromonas* SAGs; the functional proteins encoded by this category of genes also shows higher amino acid sequence variation than those encoded by other gene sets within the *Roseobacter* SAGs. We also found that genes involved in cell wall/membrane/envelope biogenesis, transcriptional regulation, and metal transport may be important for the adaptation of hadal *Roseobacter* and *Alteromonas* lineages. These results imply that the modification of cell surface-related proteins and transporters is the major direction of genomic evolution in *Roseobacter* and *Alteromonas* bacteria adapting to the hadal environment, and that prophages and transposons may be the key factors driving this process.

## Introduction

The hadal zone constitutes the deepest 45% of the vertical depth gradient of oceans (6000–11000 m), and corresponds to the highest hydrostatic pressure within the seawater environment (60–110 MPa) (Jamieson et al., 2009). Certain physical and geochemical parameters of hadal trenches—such as temperature, dissolved oxygen, and nutrients—are similar to those in abyssal plains (4000–6000 m) (Jamieson et al., 2009; Nunoura et al., 2015); however, the microbial communities of hadal trench environments are distinct from those in the abyssal environments above (Nunoura et al., 2015). Furthermore, the gene composition of trench-adapted microorganisms is distinct from that of their shallower deep-sea relatives (León-Zayas et al., 2015).

Based on 16S rRNA gene analysis and metagenomic sequencing, heterotrophic Proteobacteria have been found to be abundant and often dominant in hadal ocean zones (Nunoura et al., 2015, 2016; Liu et al., 2019). In particular, the orders *Rhodobacterales* and *Alteromonadales* are representative bacterial populations that have high relative abundances in the Challenger Deep of the Mariana Trench (Liu et al., 2019), and previous studies have shown that *Rhodobacterales* bacteria are abundant within particles in the deep sea (Eloe et al., 2011c; Li et al., 2015). Within the *Rhodobacterales*, group *Roseobacter* is a diverse alphaproteobacterial lineage whose members share > 89% identity of the 16S rRNA gene and include > 70 validly named genera and > 170 validly named species (Simon et al., 2017). This group is found ubiquitously in marine environments, accounting for up to 25% of microbial communities in coastal oceans (Buchan et al., 2005). However, genomic information from roseobacters inhabiting hadal trenches is scarce. Currently, a single-cell amplified genome (SAG) belonging to the genus *Marinosulfonomonas* recovered from the Puerto Rico Trench (PRT) is the only genomic data from roseobacters in hadal trench environments (León-Zayas et al., 2015). The 16S rRNA gene sequence from this SAG was closely related to the *Roseobacter* strain PRT1—a piezophilic strain previously isolated from the PRT (Eloe et al., 2011b)—and clustered within a phylogenetic clade of deep-sea environmental sequences. Notably, comparative genomic analyses suggested that many of the genes unique to this hadal *Marinosulfonomonas* lineage were of unknown function (León-Zayas et al., 2015).

Bacteria in the genus *Alteromonas* are primarily abundant in surface waters and are considered to be r-strategists due to their ability to grow rapidly when substrates are available (López-López et al., 2005). The isolation of deep-sea *Alteromonas* bacteria, particularly from hydrothermal vents, was carried out in the past because these bacteria have industrial applications in polymer production (Raguenes et al., 1996, 1997). *Alteromonas macleodii* is a representative species of the genus (Baumann et al., 1972), and the *A. macleodii* “deep ecotype” was recently defined as a new species, *A. mediterranea*, by Ivanova et al. (2015). *A. mediterranea* was reported to be the most abundant group within *Alteromonadales* in the hadopelagic zone of the Mediterranean Sea and may largely contribute to the *de novo* synthesis of organic carbon (Yakimov et al., 2014). Comparative genomic analyses indicated that the genomic islands of this “deep ecotype” are markedly different to those of the type strain *A. macleodii* ATCC 27126^T^ (Ivanova et al., 2015), and the exchange of some metabolism-related genes may occur between *A. mediterranea* and *A. macleodii* via mobile genetic elements (Koch et al., 2020). With the exception of this report however, genomic information from deep-sea *A. macleodii* is scarce.

The paucity of genomic data from hadal *Roseobacter* and *Alteromonas* limits our understanding of their phylogenetic traits and metabolic functions. Besides, obtaining cultured isolates form abyssal and hadopelagic environment is difficult (Eloe et al., 2011b). It is therefore necessary to obtain genomes from hadal trenches via culture-independent methods. Recently developed methods of *de novo* assembly and binning of metagenomic reads have been successfully applied to increasingly complex microbiomes, and have revealed the draft genomes of many deep lineages of bacteria and archaea. However, the representation and accuracy of metagenome bins usually deteriorates at lower taxonomic levels (e.g., below family levels) (Sczyrba et al., 2017), and this method does not recover variable regions in the genomes of non-clonal populations effectively (Pachiadaki et al., 2019). Nonetheless, these problems can be solved by combining single-cell genome sequencing with cell sorting by flow cytometry, and the resulting SAGs can be used in comparative analyses with surface-associated microbial genomes. Such analyses may provide credible clues as to the metabolic modifications of hadal *Roseobacter* and *Alteromonas* groups, and insights into the ecological strategies of typical heterotrophic bacteria adapting to hadal environments.

Here, we describe ten *Roseobacter* and eight *Alteromonas* SAGs derived from seawater sampled at a depth of 8289 m within the hadal zone of the Mariana Trench. We explore their phylogenetic characteristics and vertical distribution patterns, and we summarize the potential metabolic adaptations of these two typical marine heterotrophic populations to the hadal environment—via a comparative analysis between the Mariana Trench SAGs (MT-SAGs) and genomes of the most closely related surface water strains.

## Materials and Methods

### Sample Collection, Cell Sorting, Genome Amplification, Sequencing, and Annotation

Seawater samples were collected from the hadal zone in the Mariana Trench (11°21.847′ N, 142°20.775′ E) at 8289 m in February 2017 using a self-designed acoustic-controlled full-ocean-depth water sampler (Guo et al., 2018). Triplicate samples that comprised 1 mL of seawater in a 2 mL cryotube were taken and then stored at −80°C with sterile cryoprotectant glyTE, which contains 56% molecular-grade glycerol, 11% 100 × TE (pH 8.0), and 33% deionized water.

Single-cell sorting and whole-genome multiple displacement amplification were carried out at the Bigelow Laboratory Single Cell Genomics Center as described by Stepanauskas and Sieracki (2007). Briefly, SAG libraries were constructed in a 384-well plate and cells were screened using primer sets for archaeal and bacterial SSU rRNA genes. Ten *Roseobacter* SAGs and eight *Alteromonas* SAGs were selected for whole-genome sequencing.

Illumina sequencing libraries were prepared using Nextera XT (Illumina) reagents following the manufacturer’s instructions. Then, 150 × 2 bp paired-end reads were sequenced using the NextSeq 500 platform (Illumina). The number of raw reads produced for every selected SAG was around 10 million. Reads were quality-trimmed using Trimmomatic v0.36 (Bolger et al., 2014) with the options “slidingwindow:4:15, maxinfo:40:0.9, minlen:40,” and *de novo* assembled using SPAdes v3.10.1 (Bankevich et al., 2012) with “–sc” options selected. Only contigs longer than 1000 bp (Chijiiwa et al., 2020) and with a sequencing depth greater than 5 × were retained. The completeness, contamination, and G + C content of each assembly were evaluated using CheckM v1.0.7 (Parks et al., 2015) and protein-encoding genes were identified via the Prokka annotation pipeline v1.12 (Seemann, 2014). Proteins were annotated using: Rapid Annotation using Subsystems Technology (RAST) (Brettin et al., 2015); Kyoto Encyclopedia of Genes and Genomes (Kanehisa and Goto, 2000); Cluster of Orthologous Groups (COG) (Tatusov et al., 2003); Pfam (Finn et al., 2014); TIGRFAM (Haft, 2003); and Conserved Domain Database (Marchler-Bauer et al., 2011). Carbohydrate-active enzymes (CAZymes) were annotated using hmmsearch against the dbCAN database (e-value < 1 × 10^–15^; coverage > 0.35) (Zhang H. et al., 2018).

### Phylogeny Construction

Ten MT-SAGs and 96 published reference genomes were used to generate the *Roseobacter* phylogenomic tree; eight MT-SAGs and 43 reference genomes were used for *Alteromonas*. Phylogenetic reconstruction was based on 120 bacterial ortholog families, as described in Parks et al. (2018). Gene family members were aligned at the amino acid level using MAFFT v7.222 (Katoh and Standley, 2013), and columns with gaps were deleted using trimAl v1.4 (Capella-Gutierrez et al., 2009). Trimmed alignments were then concatenated to comprise a super-alignment with 45 904 sites. Maximum likelihood trees were built using IQ-TREE v1.6.2 (Nguyen et al., 2015) and visualized using iTOL (Letunic and Bork, 2019). On the basis of these analyses, reference genomes which had an average nucleotide identity (ANI) of more than 95% to at least one of the MT-SAGs were defined as being “closely related genomes” and were used in subsequent analyses.

### Metagenomic Read Recruitment

The MT-SAGs recovered in this study, along with the closely related reference genomes, were used to recruit reads from metagenomic datasets from the Mariana Trench (Liu et al., 2019). Briefly, Mariana Trench metagenomic data of free-living (0.22–3 μm size fraction) and particle-associated (> 3 μm size fraction) microbial assemblages at 0, 4000, 9600 (free-living only), 10400, and 10500 m (Liu et al., 2019), were collected and then trimmed as described above. The trimmed reads were then mapped to our entire collection of MT-SAGs and selected genomes using bowtie v2.3.2 (Langmead and Salzberg, 2012) with the parameter “–very-sensitive-local.” Mapped reads were stored as BAM files using samtools (Li et al., 2009), and then searched back against the SAG and reference genomes using blastn with the parameters “–evalue 1e-5 –perc_identity 95 –qcov_hsp_perc 80.” Each mapped read was recruited only by the genome with which it had the highest identity. The relative abundance of each genome within each metagenomic dataset was evaluated using the RPKG (reads recruited per kilobase of genome per gigabase of metagenome) value (Sonnenschein et al., 2017). It is calculated as (Rx/MG)/Gx, where Rx is reads mapping to genome x, MG is the metagenome size, and Gx is the genome size. Hierarchical clustering of the MT-SAGs and reference genomes, based on their vertical distribution profiles, was analyzed using R statistical packages.

### Pangenomic Analysis

A pangenomic analysis was performed on the recovered MT-SAGs and closely related reference genomes. Orthogroups (gene clusters, GCs) were identified using OrthoFinder v2.2.1 (Emms and Kelly, 2015) with “–S diamond –M msa” options. Each GC was annotated with the function most frequently associated with the genes within the group (if multiple functions were equally represented, the choice of annotation was arbitrary). The table of the distribution of GCs across all genomes was imported into Anvio5 for visualizing the comparative pangenomic analysis using the “anvi-interactive” command according to the tutorial (Eren et al., 2015).

### Analysis of Amino Acid Sequence Variation

To determine the extent of variation between orthologous proteins from the MT-SAGs and closely related genomes, MT-SAG genes that had been functionally annotated based on the COG database were selected. These genes were then translated and compared with the translated gene sequences from closely related reference genomes using blastp with an E value threshold of < 10^–5^. The percentage identity of the top hit of each queried sequence was collected, and the genes were then ranked based on dissimilarity of amino acid sequence. This ranked list of genes was divided equally into ten intervals, and the relative abundances of the genes in each COG category were calculated for each interval. Pearson correlation between the relative abundance of genes in each COG category, and the average dissimilarity of amino acid sequences to top hits, was then tested using R statistical packages.

### Codon Usage Analysis

The effective number of codons (ENC) and the G + C content of the third codon position (GC3) were calculated with CodonW v1.4.2^1^. The plot of ENC vs. GC3 was generated using the R program according to Wright (1990).

## Results and Discussion

### Genomic Properties and Phylogeny of the *Roseobacter* and *Alteromonas* MT-SAGs

Ten SAGs (R_SAG1–10) belonging to the *Roseobacter* group and eight SAGs (A_SAG1–8) belonging to the genus *Alteromonas* were recovered from water samples taken in the hadal zone of the Mariana Trench (Table 1). The assembly sizes of the *Roseobacter* SAGs ranged from 0.9 Mb to 3.8 Mb, genome completeness ranged from 21.6% to 81.2%, and G + C content ranged from 57.9% to 60.4%. The assembled *Alteromonas* SAGs ranged in size from 1.1 Mb to 4.1 Mb, genome completeness ranged from 19.8% to 88.7%, and G + C content ranged from 44.3% to 44.7%. Most cultured members of *Roseobacter* have a G + C content of 60 ± 4% (Luo et al., 2014), which is similar to the G + C range of the *Roseobacter* SAGs recovered in this study. In contrast, most uncultured free-living roseobacters derived from the surface of the ocean have been reported to have more streamlined genomes (2.5–3.5 Mbp) and lower G + C content (ca. 40%) compared with cultured strains (Luo et al., 2014). Previous metagenomic analyses have indicated that the genomic G + C content of roseobacters increases with ocean depth. This was speculated to be driven by the decreasing carbon and increasing nitrogen content of the surrounding environment (Mende et al., 2017)—because a G + C pair uses an extra nitrogen compared with an A + T pair (Hellweger et al., 2018) and high-G + C genomes tend to encode proteomes containing more nitrogen and less carbon (Bragg and Hyder, 2004; Baudouin-Cornu et al., 2004; Grzymski and Dussaq, 2012). As such, the hadal environment may facilitate a relatively higher G + C content in *Roseobacter* genomes, indeed, a roseobacter SAG derived from the PRT had a G + C content of 52% (León-Zayas et al., 2015). Unlike roseobacters, *Alteromonas* bacteria have a relatively stable G + C content (between 38% and 50%) (Mikhailov et al., 2006), and comparative analysis has shown that shallow and deep ecotypes of *Alteromonas macleodii-*related strains have a similar G + C content (from 44.6% to 44.9%) (Ivars-Martinez et al., 2008).

**TABLE 1:** Genomic characteristics of the *Roseobacter* and *Alteromonas* SAGs recovered from the Mariana Trench*^a^.*

Name	Assembly size (bp)	Completeness(%)	Contamination (%)	G + C content (%)	Coding density (%)	Number of genes
R_SAG1	2,130,175	50.17	0.35	59.8	89.12	2,134
R_SAG2	3,118,151	56.93	7.18	59.5	89.60	3,263
R_SAG3	3,766,770	81.16	0	58.9	89.18	3,649
R_SAG4	1,678,914	21.55	0	58.7	88.66	1,692
R_SAG5	2,051,189	42.66	0.18	58.5	89.40	2,011
R_SAG6	2,003,974	29.31	0	58.0	89.17	1,961
R_SAG7	1,322,719	25.72	0	59.9	88.89	1,349
R_SAG8	2,478,973	72.01	0	60.4	90.88	2,433
R_SAG9	1,379,525	32.76	0	57.9	90.86	1,369
R_SAG10	902,978	23.03	0	58.9	87.00	957
A_SAG1	3,178,530	63.98	0	44.6	87.84	2,824
A_SAG2	2,912,339	66.85	0	44.6	88.05	2,617
A_SAG3	4,099,278	88.70	0.51	44.5	88.26	3,573
A_SAG4	3,384,298	75.43	0.17	44.7	88.80	2,988
A_SAG5	1,717,477	34.48	0	44.3	87.99	1,583
A_SAG6	3,147,980	69.13	0.17	44.7	88.68	2,791
A_SAG7	2,527,570	51.67	0.34	44.6	88.26	2,254
A_SAG8	1,118,423	19.83	0	44.6	87.55	1,027

**^a^*The assembly size, completeness, contamination, and G + C content were obtained from CheckM software (Parks et al., 2015). Protein-encoding genes were identified with the Prokka annotation pipeline v1.12 (Seemann, 2014).*

**FIGURE 1:**
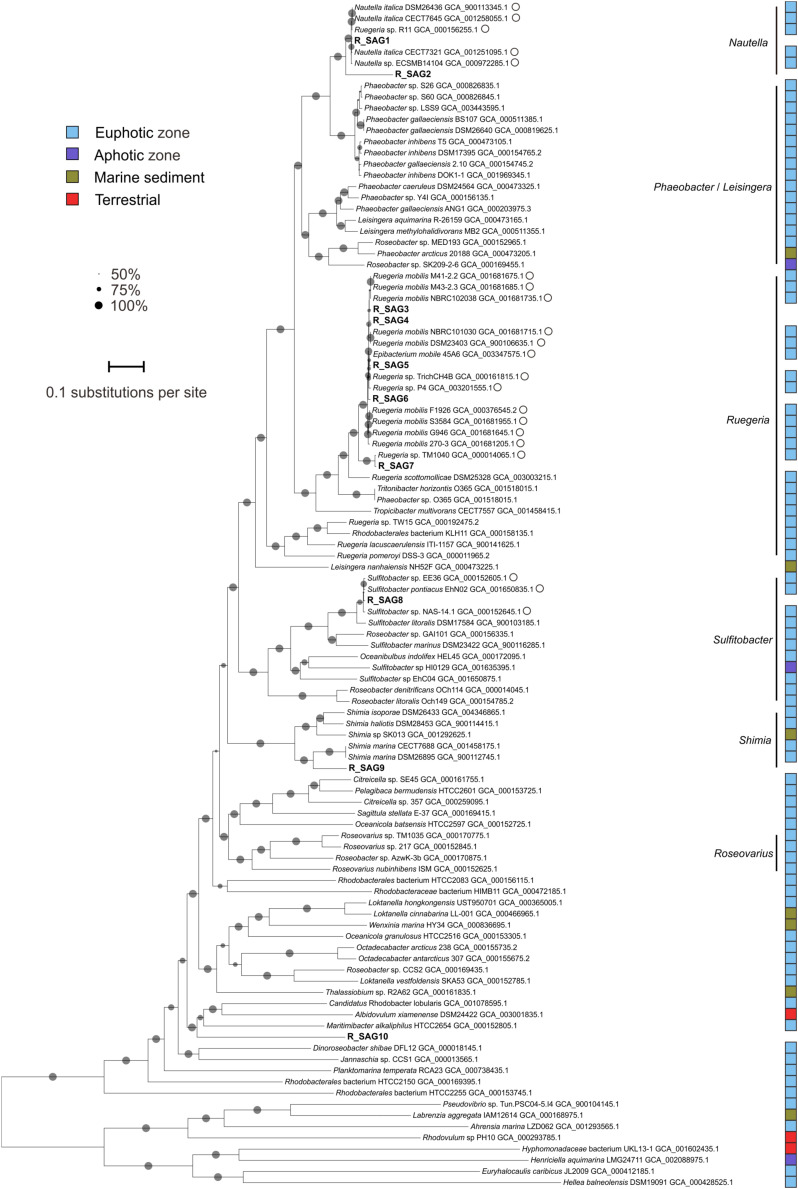
Maximum-likelihood phylogenetic tree of *Roseobacter* genomes based on the amino acid sequences of 120 bacterial orthologs. The ten SAGs from this study are shown in bold. GenBank accession numbers are shown for the reference genomes. Closely related genomes with an average nucleotide identity > 95% to at least one hadal SAG from the Mariana Trench are marked with open circles. Reference genomes from different niches are marked with colored squares, as identified in the legend inset. Bootstrap values > 50% are represented in the tree by gray circles; the size of the circle is proportional to the bootstrap value. The scale bar represents the number of amino acid substitutions per site.

The *Roseobacter* SAGs that we recovered from the Mariana Trench were phylogenetically distinct (< 85% ANI), except for R_SAG1 and R_SAG2 (95.4%) and R_SAG3–6 (96.7–97.4%) (Figure 1). R_SAG1 and R_SAG2 clustered with genomes affiliated with the genus *Nautella*. R_SAG3–6 and R_SAG7 clustered with reference genomes belonging to the genus *Ruegeria*, most of which were grouped in a clade with *Ruegeria mobilis*—a roseobacter that has a global distribution primarily in the upper ocean (Sonnenschein et al., 2017). R_SAG8 was closely related to genomes belonging to the genus *Sulfitobacter*, and had the highest identity with strain EhN02 (97.8%) isolated from an *Emiliania huxleyi* culture from the South Pacific. R_SAG9 and R_SAG10 branched distantly from the reference genomes used here. R_SAG9 was closely related to the genus *Shimia*, and had the highest identity with strain DSM 26895 (77.8%) isolated from a biofilm at a coastal fish farm, while R_SAG10 was most similar to *Candidatus* Rhodobacter lobularis (GCA_001078595.1) (72.7%), derived from the Mediterranean sponge *Oscarella lobularis*. Notably, none of the reference genomes most closely related to the MT-SAGs derive from abyssal or hadal environments (Figure 1). The leakproofness and reliability of the self-designed sampler were well testified in the previous study (Guo et al., 2018) and the recovered SAGs showed many typical traits that were widely found in deep-sea (the enrichments of prophages and transposons, heavy metal-related genes, cell wall/membrane/envelope biogenesis related genes, and nitrate reductase genes in the unique gene pools; see below). We think the SAGs are not from contaminants elsewhere in the water column. To date, little is known about the precise taxonomy of hadal roseobacters. One report from Eloe et al. (2011b) described a psychropiezophilic *Roseobacter* strain (PRT1) from 8350 m in the PRT. This strain was found to be closely related to a surface-ocean *Marinosulfonomonas* species and the widely distributed abundant marine cluster NAC11-7 (Buchan et al., 2005), according to a 16S rRNA gene-based phylogeny (Eloe et al., 2011b). Our results also suggest that hadal roseobacters could derive from the upper ocean, and contribute additional phylogenetic information to our understanding of hadal roseobacter taxonomy.

**FIGURE 2:**
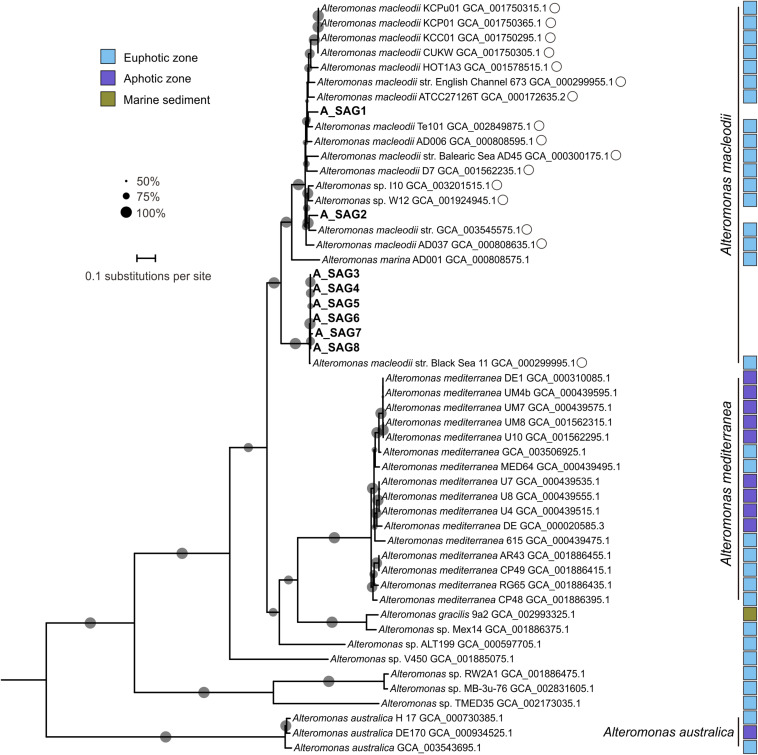
Maximum-likelihood phylogenetic tree of the *Alteromonas* genomes based on amino acid sequences of 120 bacterial orthologs. The eight SAGs from this study are shown in bold. GenBank accession numbers are shown for the reference genomes. Closely related genomes with an average nucleotide identity > 95% to at least one hadal SAG from the Mariana Trench are marked with open circles. Reference genomes from different niches are marked with colored squares, as identified in the legend inset. Bootstrap values > 50% are represented in the tree with gray circles; the size of the circle is proportional to the bootstrap value. The scale bar represents the number of amino acid substitutions per site.

All eight of the recovered *Alteromonas* SAGs clustered with genomes of *Alteromonas macleodii* strains in two separate clades: A_SAG1 and A_SAG2 (97.8% ANI), and A_SAG3–8 (99.5–100%) with high identity (99.4–99.7%) with an *A. macleodii* strain from the surface water of the Black Sea (GCA 000299995.1) (Figure 2). These clades were phylogenetically distant from the clusters of *A. mediterranea* and *A. australica*-related genomes. *A. australica* is distributed from the surface to depths of oceans, for example, it has been isolated from the surface water of the Tasman Sea (Ivanova et al., 2013) and from a depth of 1000 m in the South Adriatic (López-Pérez et al., 2014). *A. mediterranea* is a well-studied deep-sea adapted species that was first found in deep waters of the Mediterranean Sea (López-López et al., 2005) and is closely related to *Alteromonas macleodii*. Indeed for many years, *A. mediterranea* was identified as a “deep ecotype” within *A. macleodii* (López-López et al., 2005; Ivars-Martinez et al., 2008; López-Pérez et al., 2013; Ivanova et al., 2015). The unexpected absence of abyssal *Alteromonas* SAGs in our study (Figure 2) suggests that there is a marked difference between the niches of the abyssal zone and hadal trenches. We speculate that the input of organic matter associated with trench geomorphology (Nunoura et al., 2015) might facilitate *A. macleodii* blooms, because *A. macleodii* is a copiotrophic r-strategist. In addition, *A. macleodii* is considered to be mesophilic (growing between 10°C and 45°C), and to be mostly found in warm water masses (López-Pérez et al., 2014). As such, our recovery of *A. macleodii*-affiliated SAGs from the cold environ of a hadal trench extends our knowledge of temperature adaptation in this species. Similarly, a previous study by Li et al. (2015) retrieved 16S rRNA gene sequences affiliated with *A. macleodii* from a depth of 1500 m in the South China Sea.

### Vertical Distribution of the MT-SAGs and Related Reference Genomes

**FIGURE 3:**
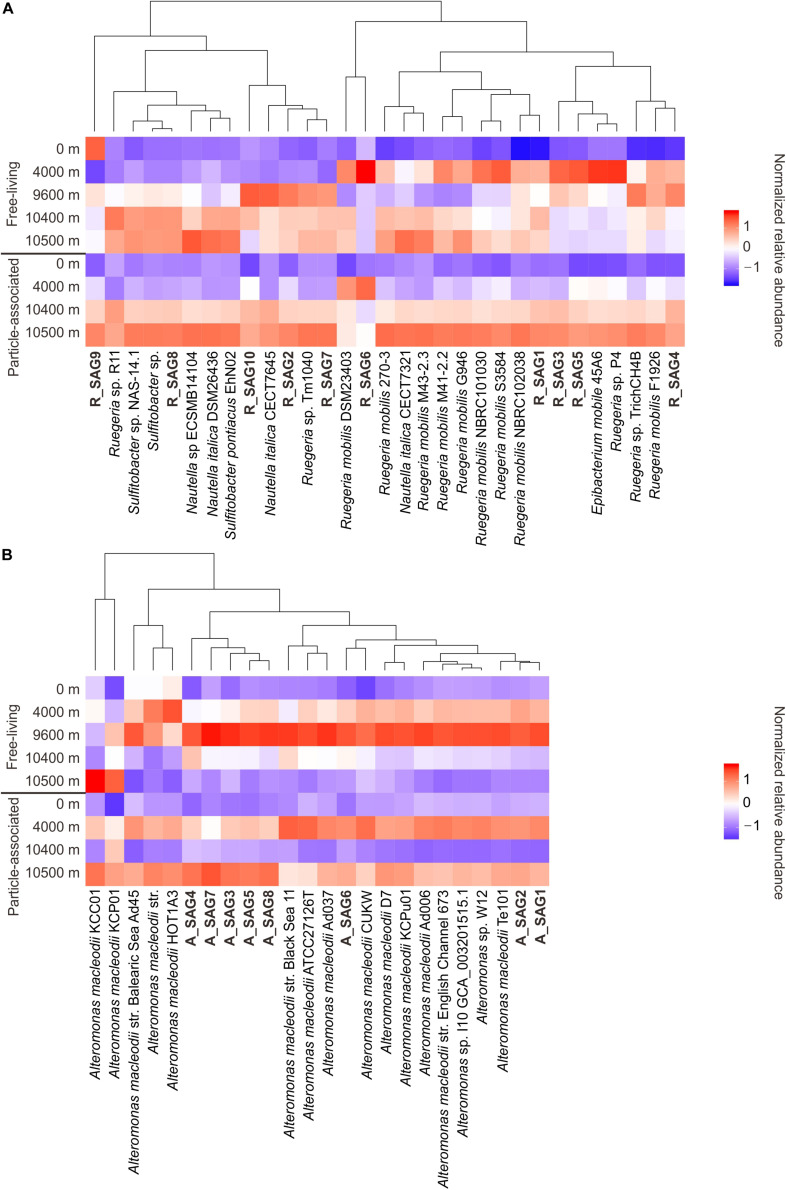
Vertical distribution of **(A)** the hadal *Roseobacter* SAGs and **(B)** the hadal *Alteromonas* SAGs, along with the closely related reference genomes. Distribution is based on genomic recruitment against the metagenomes of free-living and particle-associated microbial assemblages from the surface to depths of the Mariana Trench (Liu et al., 2019). For each size fraction, the normalized relative abundance (RPKG value) of each genome (see Methods) along depth profile was *Z*-normalized (Magalhães et al., 2008) for a clearly visible display. The SAGs from this study are shown in bold.

To clarify the depth profiles of the MT-SAGs, we carried out a recruitment analysis of the 18 recovered hadal SAGs and 37 closely related reference genomes (> 95% ANI to at least one MT-SAG) from the epipelagic zone (Figures 1, 2), against the metagenomes of free-living and particle-associated microbial assemblages from the surface to depths of the Mariana Trench (Liu et al., 2019). These depths span epipelagic zone, abyssopelagic zone, hadalpelagic zone, and near bottom zone, representing major vertical niches in the ocean. Notably, the results of this analysis showed that the recovered MT-SAGs generally had similar depth distribution patterns to closely related genomes, i.e., a higher relative abundance in abyssal or hadal waters compared with surface water (Figure 3). Within the free-living fraction, the 31 *Roseobacter* genomes (10 MT-SAGs and 21 reference genomes) mainly showed two distribution patterns (Figure 3A); their higher relative abundances were generally either recruited from the abyssal (4000 m) or hadal zone (9600, 10400, and 10500 m) with the exception of R_SAG9 that was relatively abundant in the surface water (0 m). Within the particle-associated fraction, almost all genomes showed higher relative abundances in the hadal zone (10400 and 10500 m), particularly at 10500 m. This conflicts with a previous report that blasted trench metagenomic reads against the NCBI-nr database and found that the relative abundance of reads affiliated with *Rhodobacterales* was highest in the surface water and decreased with depth (Liu et al., 2019). These contrasting trends imply that our recovered hadal *Roseobacter* SAGs and closely related genomes may represent deep-sea adapted strains within the order *Rhodobacterales*. This is consistent with another study; it reported that the *Ruegeria mobilis* strain NBRC101030, to which R_SAG3–6 is closely related (Figure 1), might prefer the low temperature and high nitrate concentration of deep ocean environments (Sonnenschein et al., 2017).

All of the free-living *Alteromonas* genomes (including 8 MT-SAGs and 16 reference genomes of *A. macleodii*) had their highest relative abundances in the metagenomes from 9600 m, followed by 4000 m—except for four reference genomes whose relative abundances were highest either in the metagenomes from 4000 m or 10500 m (Figure 3B). These patterns are consistent with a depth profile of *Alteromonadales* based on trench metagenome annotation by the NCBI-nr database, in which the relative abundance of *Alteromonadales* increases from the surface water to the hadal zone and then sharply decreases near the trench bottom (Liu et al., 2019). It has been speculated that an enrichment of alkane-degrading bacteria such as *Oceanospirillales* can restrict the niche of *Alteromonadales* near the trench bottom (Liu et al., 2019). However in our analysis, all of the particle-associated genomes had their highest relative abundances in the metagenomes from 4000 m and 10500 m (Figure 3B). We infer that the particle-associated *A. macleodii* blooms at 10500 m are probably facilitated by the resuspension of organic particles at the bottom of hadal trench.

To further clarify whether these MT-SAG lineages prefer more particle-associated lifestyle compared with their surface relatives, we searched for CAZymes genes in the MT-SAGs and closely related reference genomes, which are involved in the degradation of carbohydrates and linked to particle-associated prokaryotes (Zhao et al., 2020). While abundant CAZymes genes were found in the MT-SAGs (Supplementary Table S1), there were no significant differences in coding density and class composition of CAZymes genes between the MT-SAGs and their surface relatives (Supplementary Figure S1 and Supplementary Table S1). This indicates that the particle-associated lifestyle is not a unique trait for these hadal *Roseobacter* and *Alteromonas* lineages compared with the shallow-water relatives.

### Genomic Adaptation of MT-SAG Lineages to Hadal Environments

**FIGURE 4:**
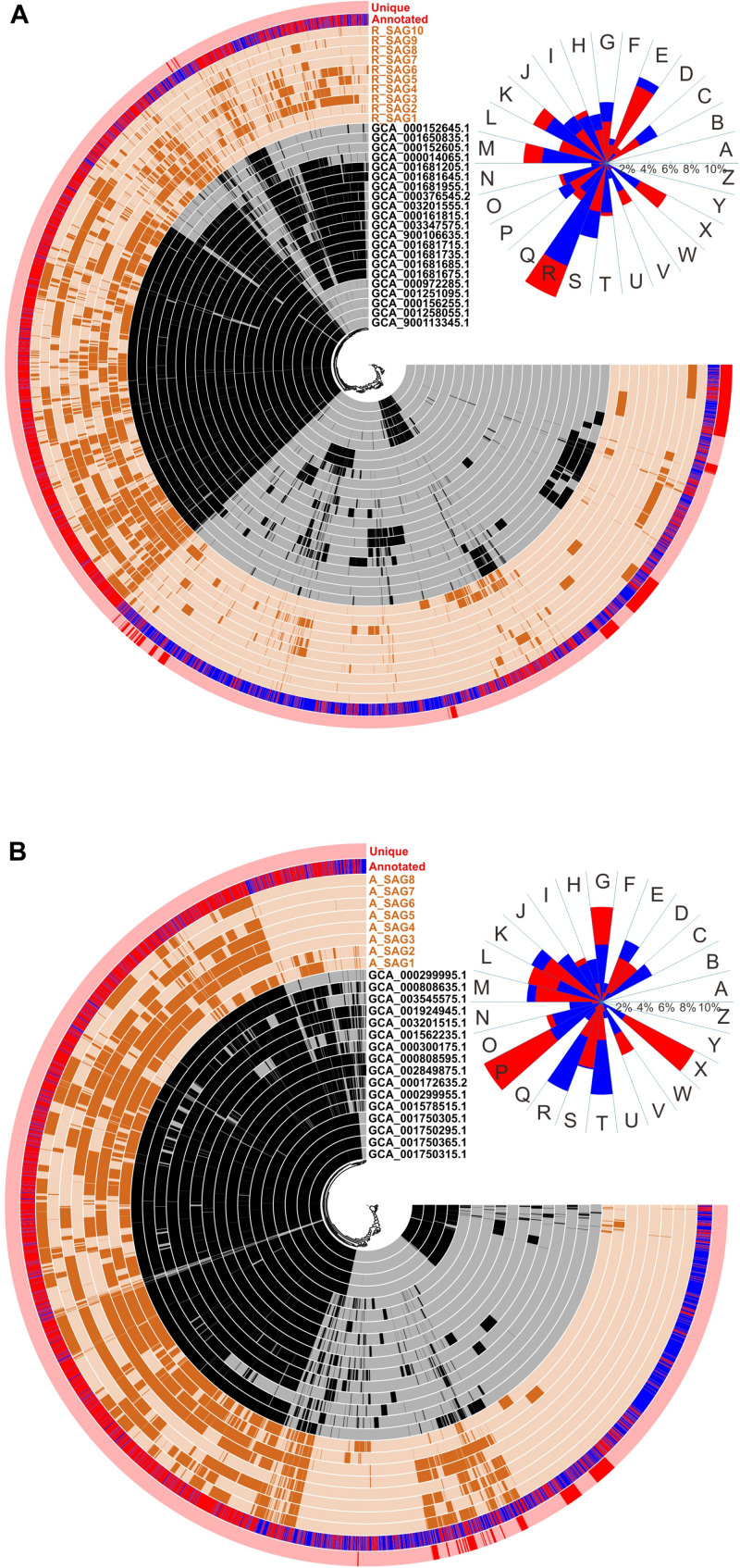
Comparative pangenomic analysis between **(A)** the hadal *Roseobacter* SAGs or **(B)** the hadal *Alteromonas* SAGs, and the closely related reference genomes. The SAGs from this study are represented in outer circles in brown; the closely related genomes are represented in inner circles in black. Red bars in the outermost circle denote GCs unique to the hadal SAGs compared with the closely related genomes. The second most outer circle displays the COG annotation of GCs (genes with known functions are shown in red; unknown are in blue). The gene clusters are arrayed based on the hierarchical clustering (the center of each panel) of their distribution across all genomes. At the upper right corner of each panel, the functional composition of all annotated GCs (blue sectors) and GCs unique to the hadal SAGs (red sectors) are shown, based on the following COG categories: A, RNA processing and modification; B, chromatin structure and dynamics; C, energy production and conversion; D, cell cycle control, cell division, chromosome partitioning; E, amino acid transport and metabolism; F, nucleotide transport and metabolism; G, carbohydrate transport and metabolism; H, coenzyme transport and metabolism; I, lipid transport and metabolism; J, translation, ribosomal structure and biogenesis; K, transcription; L, replication, recombination and repair; M, cell wall/membrane/envelope biogenesis; N, cell motility; O, posttranslational modification, protein turnover, chaperones; P, inorganic ion transport and metabolism; Q, secondary metabolites biosynthesis, transport and catabolism; R, general function prediction only; S, function unknown; T, signal transduction mechanisms; U, intracellular trafficking, secretion, and vesicular transport; V, defense mechanisms; W, extracellular structures; X, mobilome: prophages, transposons; Y, nuclear structure; Z, cytoskeleton. The blue and red sectors overlap each other and the shorter ones are over the longer ones.

To identify adaptations of the MT-SAGs to the hadal environment, we carried out pangenomic analyses of the recovered *Roseobacter* and *Alteromonas* SAGs and the closely related reference genomes (Figure 4). These revealed that the *Alteromonas* genomes contained a higher proportion of conserved GCs than the *Roseobacter* genomes (Figure 4), which could be caused by the higher identity among the former. Overall, 59.6% and 64.0% of the GCs in the *Roseobacter* and *Alteromonas* genomes were annotated by the COG database, respectively. We also compared the functional composition of the GCs unique to the MT-SAGs with the total pool of GCs, based on COG annotation. The *Roseobacter* MT-SAGs contained 755 unique GCs, among which only 253 were annotated; these were distributed across 22 functional categories. The GCs assigned a ‘general function prediction only’ (category R) were most abundant, accounting for 13.5% of the 253 unique GCs and 10.3% of the total annotated GCs (Figure 4A). These data concur with a previous report from the Mediterranean Sea (León-Zayas et al., 2015), and suggest that some unknown functions may be important for the adaptation of *Roseobacter* lineages to hadal environments. In addition, GCs involved in ‘cell wall/membrane/envelope biogenesis’ (category M), ‘transcription’ (category K), and ‘mobilome: prophages, transposons’ (category X), accounted for a higher proportion of the 253 unique GCs (7.8, 7.8, and 6.4%, respectively) than of the total annotated GCs (6.1, 6.5, and 3.6%, respectively). The *Alteromonas* MT-SAGs contained 279 unique GCs, 96 of which were annotated by COG and distributed across 21 categories (Figure 4B). GCs involved in ‘inorganic ion transport and metabolism’ (category P), ‘mobilome: prophages, transposons’ (category X), ‘carbohydrate transport and metabolism’ (category G), and ‘replication, recombination and repair’ (category L), accounted for a higher proportion (12.5, 9.8, 8.9, and 7.1%, respectively) of the 96 unique GCs than of the total annotated GCs (5.5, 2.5, 5.4, and 4.4%, respectively) (Figure 4B).

**FIGURE 5:**
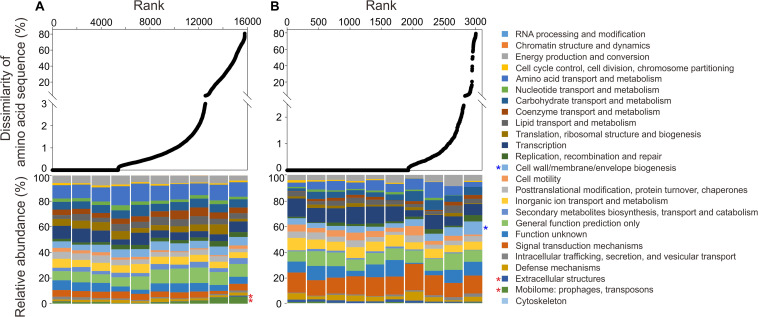
The rank dissimilarity of amino acid sequence to top hit of annotated genes from **(A)** the hadal *Roseobacter* SAGs and **(B)** the hadal *Alteromonas* SAGs, blasted against the translated gene sequences from closely related reference genomes. The corresponding functional composition (based on COG categories) of ten intervals of the ranked genes is shown in the lower panel. Significantly positive correlations (Pearson correlation test, *p* < 0.01) between the relative abundance of a functional category and amino acid sequence variation are marked with red asterisks (for *Roseobacter*) and blue asterisks (for *Alteromonas*). The order of the colors is the same between the legend and the chart.

To further clarify amino acid sequence variation of the recovered MT-SAGs compared with the reference genomes, the annotated genes of the MT-SAGs were translated and blasted against the translated gene sequences from reference genomes using blastp. The rank dissimilarity of amino acid sequence to top hit, and the corresponding functional composition of COG categories over ten intervals (see section “MATERIALS AND METHODS”), are shown in Figure 5. For the *Roseobacter* SAGs, the proportion of genes involved in ‘extracellular structure’ (category W) and ‘mobilome: prophages, transposons’ (category X) exhibited a highly significant positive correlation with amino acid sequence variation (*p* = 0.00015 and 0.0012, respectively); for the *Alteromonas* SAGs, the relative abundance of genes involved in ‘cell wall/membrane/envelope biogenesis’ (category M) was highly significantly positively correlated with amino acid sequence variation (*p* = 0.0012).

To further clarify whether these overrepresented unique genes could express functions or only originate from aimless random mutations, the codon usage analysis was performed to obtain the ENC and GC3 values. ENC value ranges between 20 (one codon is exclusively used for each amino acid) and 61 (the use of alternative synonymous codons is equally likely; Wright, 1990). A lower ENC value represents a higher codon bias, which can infer higher expression activity of genes (Wright, 1990). Our results showed that the average ENC values of unique genes within most COG categories were relatively higher than those of all genes (Supplementary Figure S2), implying these unique genes have relatively lower expression activity compared with most core genes. The main features of codon usage patterns for the unique and all genes were further analyzed by plotting ENC versus GC3 (Wright, 1990). The data dots will lie on the expected curve of ENC versus GC3 under random codon usage, when no selection effects, or below the curve when selective constraints on codon bias. A majority of these overrepresented unique genes were lying well below the expected curve of ENC versus GC3 under random codon usage (Supplementary Figures S3A,B), suggesting that some selection processes rather than random mutations influenced the codon usage variation among these unique genes. Similarly, most genes of the total gene set do not have random codon usage (Supplementary Figures S3C,D). The basically consistent ranges of ENC and GC3 values between the unique genes and the total gene set indicate that the unique genes generally were not acquired from exogenous sources. These analyses suggest that these overrepresented unique genes could have the potentials to play roles in hadal environments rather than originate from random mutations.

Taken together, our pangenomic and amino acid sequence variation analyses suggest that genes associated with ‘mobilome: prophages, transposons’ (category X) and ‘cell wall/membrane/envelope biogenesis’ (category M) are significantly related to the hadal niche. Besides, genes involved in transcription and inorganic ion transport might be important for the adaptation of *Roseobacter* and *Alteromonas* lineages to the hadal zone.

### Cell Membrane and Extracellular Structure

The unique GCs involved in ‘cell wall/membrane/envelope biogenesis’ (category M) from the *Roseobacter* and *Alteromonas* MT-SAGs were mostly related to polysaccharide synthesis and modification (Supplementary Tables S2, S3), e.g., glycosyltransferase and UDP-N-acetylglucosamine 2-epimerase (Campbell et al., 2000; Mesleh et al., 2016). Polysaccharides are the main structural component of cell-walls and we speculate that roseobacters need compensatory cell wall synthesis to adapt to hadal environments, due to the mechanical damage caused to cell walls by high hydrostatic pressure (Wang et al., 2013). Polysaccharides are also a major component of extracellular polymers and the matrix of biofilms, which can alter the physical and biogeochemical microenvironments that surround cells. By providing adhesion, protection, and structure, such molecules protect cells against harsh conditions—e.g., low temperature, high hydrostatic pressure, and heavy metals (Nichols et al., 2005; Limoli et al., 2015; Breider et al., 2019). For instance, due to their efficient adsorption of heavy metals (Iyer et al., 2005), certain polysaccharides may play an important role in bacterial resistance to heavy metals in deep-sea hydrothermal vents (Nichols et al., 2005). In addition, extracellular polysaccharides can promote the cell’s defense against viral attack (Deming and Young, 2017), given a potentially higher impact of viruses on cells in the hadal zone (the increased representation of the COG category X genes; see below).

Several of the unique *Roseobacter* MT-SAG GCs within category M were related to lipoprotein-sorting proteins, channel proteins, and ABC-type transporters (Supplementary Table S2). Lipoprotein-sorting proteins play vital roles in the biogenesis of the outer membrane through correctly sorting other lipoproteins to the outer membrane (Okuda and Tokuda, 2011). Mechanosensitive channels respond to pressure inside a cell by sensing changes in the properties of the membrane, and might also have roles during cell-wall remodeling (Booth et al., 2007). Thus, the unique GCs associated with membrane biogenesis and channel proteins suggest potential mechanisms by which the Mariana Trench lineages have adapted to high hydrostatic pressure (Macdonald and Martinac, 2005). This concurs with a previous study which also documented abundant lipoproteins in a deep-sea bacterium *Oceanobacillus iheyensis* HTE831 isolated from the Iheya ridge (Graham et al., 2007). Unique GCs in the MT-SAGs were also associated with ABC-type transporters, which are concerned with maintaining outer membrane lipid asymmetry (Supplementary Table S2). This serves to prevent phospholipids accumulating in the outer leaflet of the outer membrane and to decrease cellular sensitivity to toxic small molecules (Malinverni and Silhavy, 2009). Accordingly, the unique GCs related to ABC-type transporters in MT-SAGs might help trench lineages resist the persistent pollutants found within the Mariana Trench (Jamieson, 2018).

### Transcriptional Regulation

Genes encoding transcription regulatory factors constituted another major functional category of unique GCs (K: transcription), particularly within the *Roseobacter* MT-SAGs (Figure 4). Most of these transcriptional regulators belonged to the AcrR, AraC, ArsR, and GbsR families (Supplementary Table S2); these regulators have diverse roles. The AcrR family regulates proteins responsible for a wide range of cellular activities, including homeostasis, osmotic stress, efflux pumps, and the biosynthesis of antibiotics (Ramos et al., 2005), while AraC-related regulators have been associated with the control of catabolic pathways, e.g., degradation pathways of aromatic compounds (Tropel and van der Meer, 2004). ArsR proteins can derepress, by binding metal ions, the expression of operons responding to stress induced by heavy metal toxicity, and thus play a role in heavy metal resistance (Busenlehner et al., 2003)—especially zinc (Osman and Cavet, 2010). Last, GbsR is a member of the superfamily of MarR-type regulators, and is able to control the expression of genes involved in various metabolic pathways (Ronzheimer et al., 2018). Transcriptional regulation is considered to be one of the major response mechanisms against changing environments, and the enrichment of transcriptional regulation-related genes has been reported in metagenomes from the PRT (Eloe et al., 2011a). Similarly, a substantial portion of unique genes in hadal SAGs recovered from the PRT were assigned to the ‘transcription’ category (León-Zayas et al., 2015). Physiological experiments with piezophilc and piezosensitive bacteria have also revealed active transcriptional regulation under conditions of high hydrostatic pressure or low temperature (Ishii et al., 2005; Jian et al., 2016).

The curved DNA-binding protein A (CbpA)-encoding gene was also represented in the unique *Roseobacter* MT-SAG GCs belonging to the ‘transcription’ category (Supplementary Table S2). CbpA is a multifunctional heat shock protein that is able to stimulate the ATPase activity of DnaK—a major chaperone that is expressed in response to stress (Evans et al., 2010). Previous studies have indicated that heat shock proteins can be induced (Simonato et al., 2006) to protect cells against damage (Aertsen et al., 2004) or to maintain protein complex machinery for growth and viability at high hydrostatic pressures (Sato et al., 2015).

### Inorganic Metal Transport

An abundance of heavy metal-related genes are commonly detected in samples from hadal trench environments—such as the PRT, Yap Trench, and Hellenic Trench (Eloe et al., 2011a; Smedile et al., 2013; Zhang X. et al., 2018)—due to anthropogenic pollution and litter debris being retained in trenches by the funneling mechanism of trench topography (Jamieson, 2018). Previous studies have indicated the accumulation of heavy metals in fishes, sinking particles, and sediments from the hadal zone of the Mariana Trench (Luo et al., 2018; Welty et al., 2018; Xiao et al., 2020). Here, we also identified unique genes related to metal transport in the MT-SAGs. For example, overrepresented in the unique GCs of the *Alteromonas* MT-SAGs were genes involved in ‘inorganic ion transport and metabolism’ (category P), such as those encoding components of the Co/Zn/Cd efflux system (Supplementary Table S3). We speculate that these components could help trench lineages expel toxic heavy metals from the intracellular space, which is consistent with a previous report on the high tolerance of “deep-ecotype” *Alteromonas* to heavy metals (Ivars-Martinez et al., 2008). A further large portion of unique MT-SAGs GCs was related to nitrate and molybdate transporters (Supplementary Table S3) that are involved in nitrate respiration (Ivars-Martinez et al., 2008). Genes encoding nitrate reductase were also found among the unique GCs of the *Alteromonas* MT-SAGs, again resembling data from the “deep-ecotype” *Alteromonas* (Ivars-Martinez et al., 2008). Last, unique GCs related to the ABC-type Fe^3+^ transporter and Fe^2+^/Mn^2+^ transporter of the VIT1/CCC1 family were found in the *Roseobacter* MT-SAGs. This is significant as ABC-type transporters are reported to play an important role in iron uptake in deep-sea hydrothermal vents (Li et al., 2014), and the Mn^2+^ transporter of the VIT1/CCC1 family was reported in eukaryotic cells to be able to transport Mn^2+^ into vacuoles, thereby minimizing the toxic effects of an over-accumulation of Mn^2+^ in cells in a Mn-abundant environment (Tsednee et al., 2019). It is possible that the Mn^2+^ transporter may also play a similar role in the hadal roseobacters since some *Roseobacter* cells may contain vacuole-like structures (Nojiri et al., 2015).

### Prophages and Transposons

In both the *Roseobacter* and *Alteromonas* MT-SAGs, genes associated with prophages and transposons were found in relatively higher proportions in the unique gene pool compared with the total gene pool, and the proteins encoded by these genes within the *Roseobacter* SAGs had a higher amino acid sequence variation compared with the closely related reference genomes than other gene sets (Figures 4, 5). Similarly, the enrichment of phage integrases or transposases in deep-ocean microbes has been documented in research from the PRT (León-Zayas et al., 2015), North Pacific (DeLong et al., 2006; Konstantinidis et al., 2009), and the Mediterranean (Smedile et al., 2013), while such enrichments were also identified in some roseobacters isolated from the sea ice of the Arctic and Antarctic (Vollmers et al., 2013). This is also consistent with observations that the ratio of viruses to prokaryotes typically increases with depth (Parada et al., 2007; De Corte et al., 2010; Nunoura et al., 2015). The accumulation of phage or transposons may be important for psychrophilic bacterial genome evolution (Allen et al., 2009; Wahl et al., 2019), and the protein clusters found in viruses—such as those involved in DNA replication, DNA repair, and motility—can boost host fitness for the deep-sea environment (Hurwitz et al., 2015). We found more unique GCs related to prophages in the *Roseobacter* MT-SAGs than in the *Alteromonas* MT-SAGs, suggesting that roseobacters are more easily infected by viruses and potentially have higher genome openness than *Alteromonas* bacteria. A previous study on *Ruegeria mobilis* indicated that intra-species variation originates primarily from prophages (Sonnenschein et al., 2017). Thus, we speculate that prophages and transposons may contribute to horizontal gene transfer and evolution of hadal bacterial genomes.

In category X (mobilome: prophages, transposons), both the *Roseobacter* and *Alteromonas* MT-SAGs contained unique GCs related to plasmid stabilization system proteins (Supplementary Tables S2, S3). This indicates the presence of plasmids in these hadal *Roseobacter* and *Alteromonas* cells. We also identified the multiple genes (0-5 copies for each) involved in plasmid replication, which represents the core functions of a plasmid (Petersen et al., 2009), in these SAGs based on the RAST annotation (Supplementary Table S4). This is consistent with *Roseobacter* and *Alteromonas* bacterial genomes containing a variable number of plasmids (López-Pérez and Rodriguez-Valera, 2016; Sonnenschein et al., 2017). Moreover, previous studies indicated that the plasmid transfer may contribute to horizontal gene transfer among microbial populations (Frischer et al., 1994; Sobecky and Hazen, 2009).

## Conclusion

We recovered multiple SAGs belonging to the *Roseobacter* group and genus *Alteromonas* from the hadal zone in the Mariana Trench. Phylogenomic analyses showed that these hadal SAGs have a closer relationship to surface-associated strains despite recruitment analyses indicating that their relative abundance was mainly higher in the hadal zone. This suggests that while the MT-SAG lineages identified here may have originated from surface species, they now dwell in the hadal trench. This leads us to propose that an accumulation of organic matter associated with trench geomorphology may result in hadal trenches offering a nutritive condition that is similar to surface water and unlike the abyssal zone. Our comparative genomic analysis indicated that genes unique to the hadal SAGs were mainly involved in the mobilome (prophages and transposons), cell wall/membrane/envelope biogenesis, transcription, and inorganic ion transport. These functional categories may thus be important for the adaptation of the *Roseobacter* and *Alteromonas* lineages to the hadal trench. We speculate that prophages and transposons may contribute to the genomic evolution of hadal *Roseobacter* and *Alteromonas* through genetic recombination. Furthermore, modified transcriptional regulators may be employed to induce specific functional components, such as membrane proteins and transporters, to help cells to withstand high hydrostatic pressures, low temperatures, and toxic heavy metals in the hadal trench environment. These analyses provide insight into the potential metabolic modifications and ecological strategies used by two typical heterotrophic bacterial groups—*Roseobacter* and *Alteromonas*—to adapt to hadal environments; however, further phenotypic diagnosis of gene function (e.g., at transcription and protein levels) is needed to verify these potential adaptation mechanisms in hadal trenches.

## Data Availability Statement

The single amplified genomes datasets produced in this study can be found in online repositories. The names of the repository/repositories and accession number(s) can be found at: https://www.ncbi.nlm.nih.gov/genbank/, PRJNA613356.

## Author Contributions

YZ conceived and designed the research. MC, XF, YS, and KT analyzed the data. JT organized the cruises and provided the background data. MC, YZ, and YS wrote the manuscript. XF, NJ, KT, and JT contributed to the review and editing of the manuscript. All authors contributed to the article and approved the submitted version.

## Conflict of Interest

The authors declare that the research was conducted in the absence of any commercial or financial relationships that could be construed as a potential conflict of interest.

## Footnotes

1 http://codonw.sourceforge.net//
