# Supplementary material for: Genomic Characteristics and Potential Metabolic Adaptations of Hadal Trench Roseobacter and Alteromonas Bacteria Based on Single-Cell Genomics Analyses
Source: Front Microbiol. 2020 Jul 24;11:1739. doi: 10.3389/fmicb.2020.01739 (PMC7393951; doi:10.3389/fmicb.2020.01739)
Supplement: Supplementary file 1 [file Data_Sheet_1.docx]

***Supplementary Material for***

**Genomic Characteristics and Potential Metabolic Adaptations of Hadal Trench *Roseobacter* and *Alteromonas* Bacteria Based on Single-cell Genomics Analyses**

Mingming Chen^1†^, Yu Song^1†^, Xiaoyuan Feng^2†^, Kai Tang^1^, Nianzhi Jiao^1^, Jiwei Tian^3^, Yao Zhang^1*^

^1^State Key Laboratory of Marine Environmental Science, College of Ocean and Earth Sciences, Xiamen University, Xiamen, China

^2^School of Life Sciences, The Chinese University of Hong Kong, Hong Kong, China

^3^Physical Oceanography Laboratory, Ocean University of China, Qingdao, China

Running Title: Hadal trench single-cell genomes

^*^Correspondence: Yao Zhang ([yaozhang@xmu.edu.cn](mailto:yaozhang@xmu.edu.cn))

^†^Contributed equally


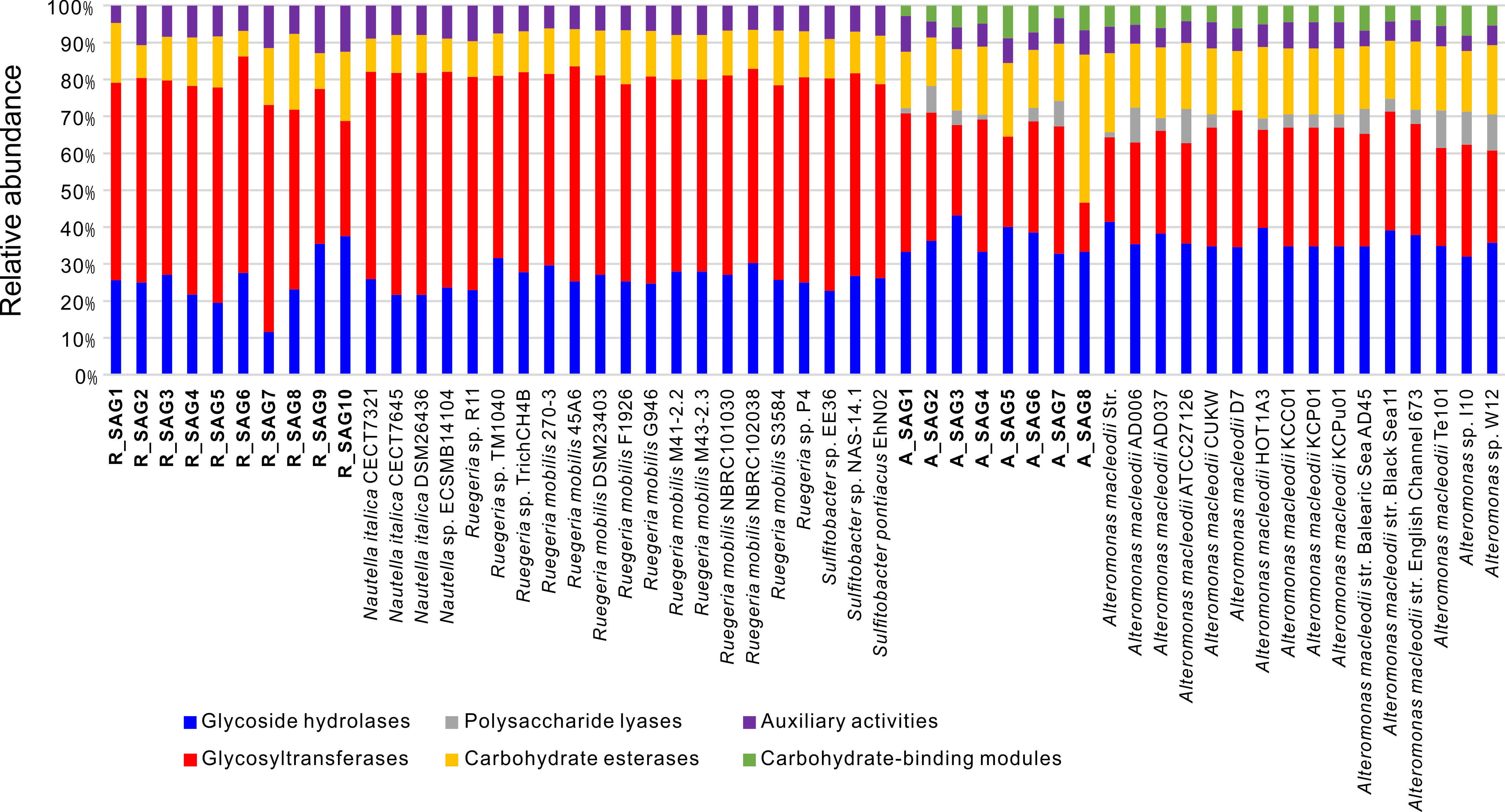


**Fig S1.** Class composition of carbohydrate-active enzymes (CAZymes) genes in the MT-SAGs and the closely related reference genomes. The SAGs from this study are shown in bold.

**
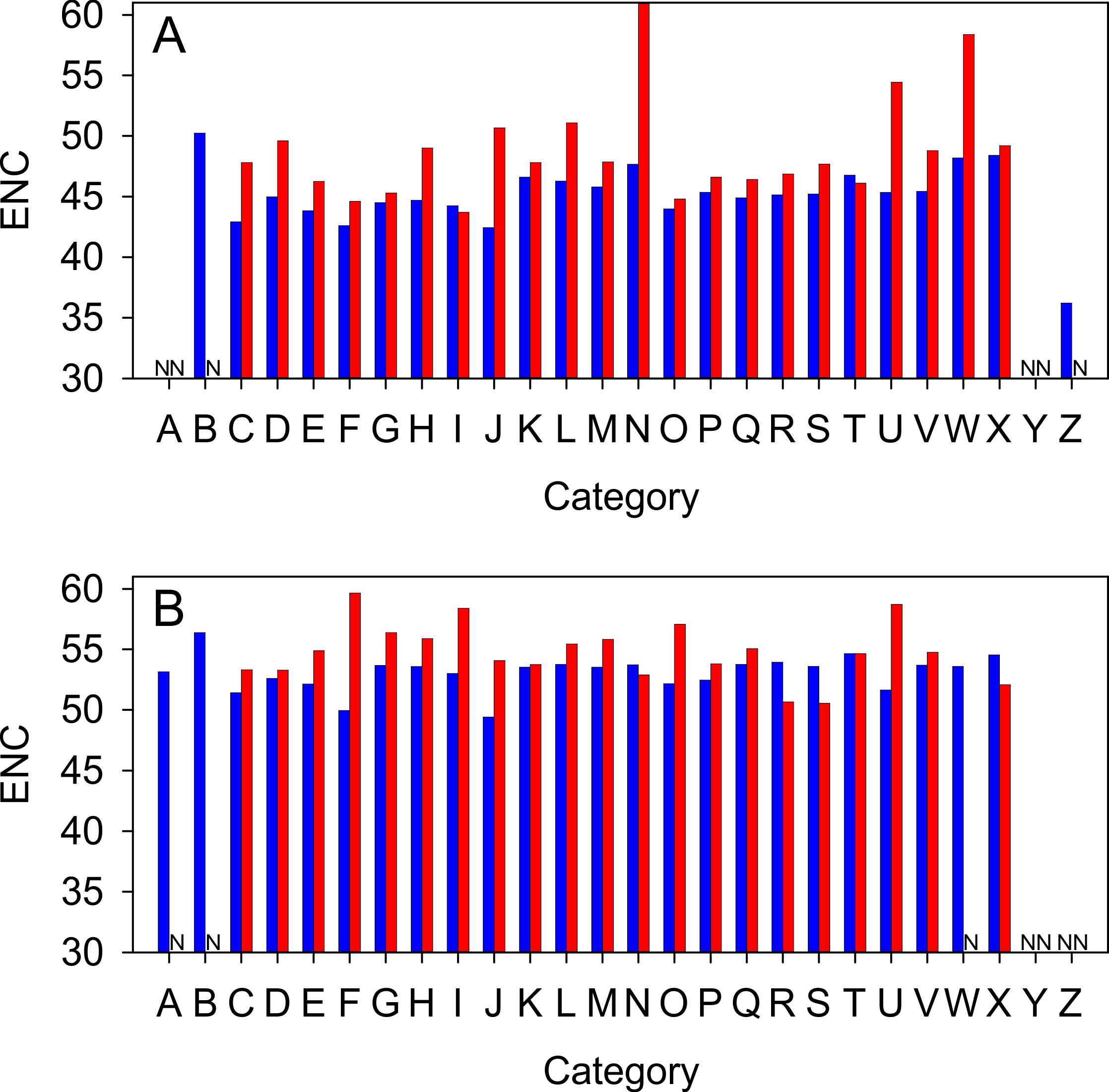
**

**Fig S2.** Distribution of the average effective number of codons (ENC) of all (blue) and unique (red) genes across different COG categories for the (A) *Roseobacter* and (B) *Alteromonas* MT-SAGs. “N” represents no data. COG category A, RNA processing and modification; B, chromatin structure and dynamics; C, energy production and conversion; D, cell cycle control, cell division, chromosome partitioning; E, amino acid transport and metabolism; F, nucleotide transport and metabolism; G, carbohydrate transport and metabolism; H, coenzyme transport and metabolism; I, lipid transport and metabolism; J, translation, ribosomal structure and biogenesis; K, transcription; L, replication, recombination and repair; M, cell wall/membrane/envelope biogenesis; N, cell motility; O, posttranslational modification, protein turnover, chaperones; P, inorganic ion transport and metabolism; Q, secondary metabolites biosynthesis, transport and catabolism; R, general function prediction only; S, function unknown; T, signal transduction mechanisms; U, intracellular trafficking, secretion, and vesicular transport; V, defense mechanisms; W, extracellular structures; X, mobilome: prophages, transposons; Y, nuclear structure; Z, cytoskeleton.

**
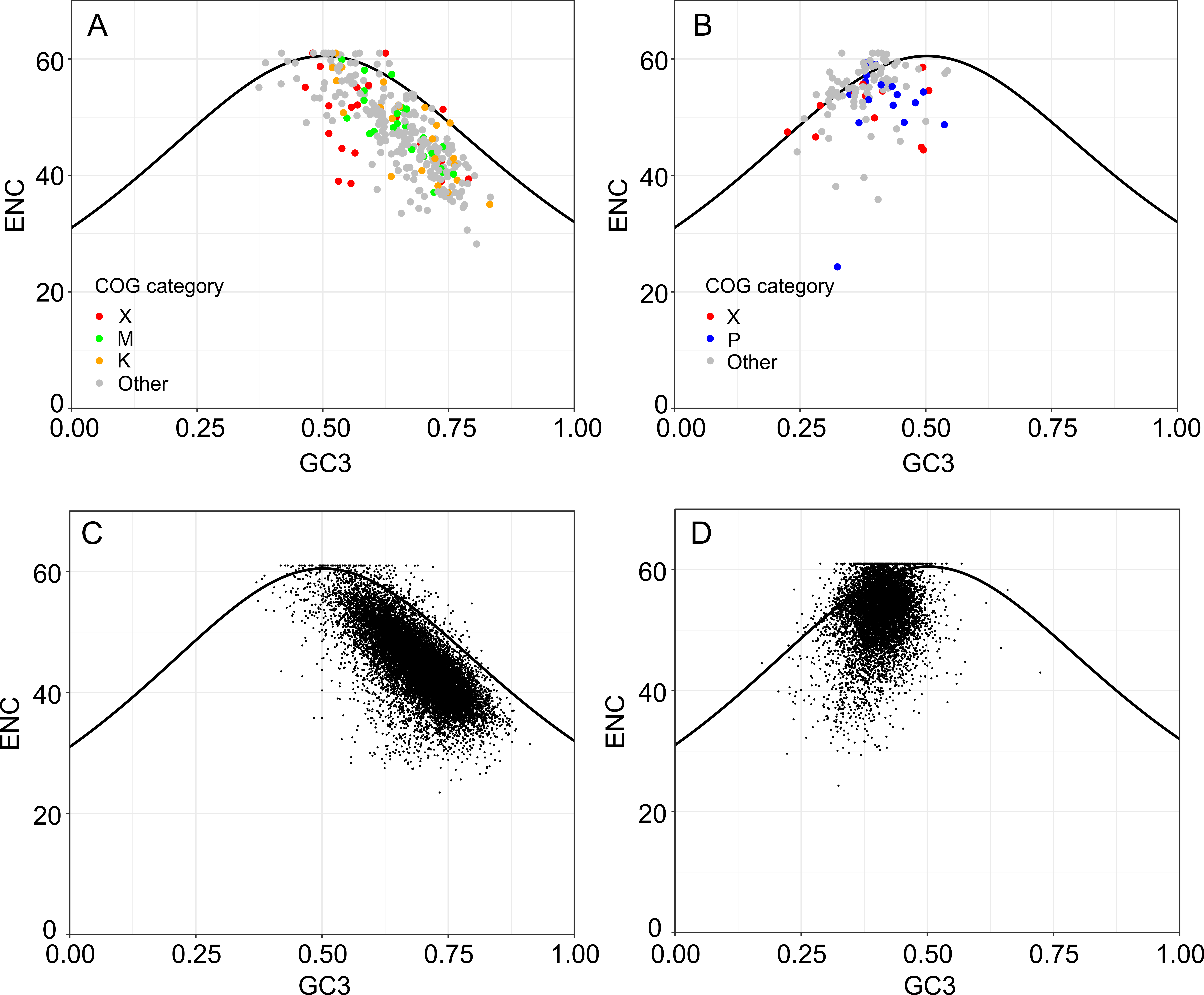
**

**Fig S3.** The plot of the effective number of codons (ENC) versus the G+C content of the third codon position (GC3) for (A and B) unique and (C and D) total genes of the (A and C) *Roseobacter* and (B and D) *Alteromonas* MT-SAGs. The continuous curve represents the expected curve of ENC versus GC3 under random codon usage. COG category X, mobilome: prophages, transposons; M, cell wall/membrane/envelope biogenesis; K, transcription; P, inorganic ion transport and metabolism.

**Table S1.** Summary of CAZymes genes in the MT-SAGs and closely related reference genomes

| Item | Accessions | Num. of CAZymes genes | Gene density of CAZymes (copies/Mbp) |
| --- | --- | --- | --- |
| R_SAG1 | GCA_012395785.1 | 41 | 19.25 |
| R_SAG2 | GCA_012395765.1 | 56 | 17.95 |
| R_SAG3 | GCA_012395745.1 | 57 | 15.12 |
| R_SAG4 | GCA_012395705.1 | 23 | 13.69 |
| R_SAG5 | GCA_012395715.1 | 33 | 16.10 |
| R_SAG6 | GCA_012395685.1 | 27 | 13.50 |
| R_SAG7 | GCA_012395655.1 | 26 | 19.70 |
| R_SAG8 | GCA_012395645.1 | 39 | 15.73 |
| R_SAG9 | GCA_012395615.1 | 31 | 22.46 |
| R_SAG10 | GCA_012395595.1 | 16 | 17.78 |
| *Nautella italica* CECT7321 | GCA_001251095.1 | 87 | 21.43 |
| *Nautella italica* CECT7645 | GCA_001258055.1 | 86 | 21.23 |
| *Nautella italica* DSM26436 | GCA_900113345.1 | 86 | 21.18 |
| *Nautella* sp. ECSMB14104 | GCA_000972285.1 | 87 | 22.25 |
| *Ruegeria* sp. R11 | GCA_000156255.1 | 81 | 21.20 |
| *Ruegeria* sp. TM1040 | GCA_000014065.1 | 77 | 18.55 |
| *Ruegeria* sp. TrichCH4B | GCA_000161815.1 | 69 | 14.71 |
| *Ruegeria mobilis* 270-3 | GCA_001681205.1 | 78 | 15.92 |
| *Ruegeria mobilis* 45A6 | GCA_003347575.1 | 76 | 15.64 |
| *Ruegeria mobilis* DSM23403 | GCA_900106635.1 | 69 | 14.65 |
| *Ruegeria mobilis* F1926 | GCA_000376545.2 | 72 | 14.91 |
| *Ruegeria mobilis* G946 | GCA_001681645.1 | 70 | 14.40 |
| *Ruegeria mobilis* M41-2.2 | GCA_001681675.1 | 72 | 14.88 |
| *Ruegeria mobilis* M43-2.3 | GCA_001681685.1 | 72 | 14.91 |
| *Ruegeria mobilis* NBRC101030 | GCA_001681715.1 | 71 | 15.04 |
| *Ruegeria mobilis* NBRC102038 | GCA_001681735.1 | 73 | 15.08 |
| *Ruegeria mobilis* S3584 | GCA_001681955.1 | 71 | 14.82 |
| *Ruegeria* sp. P4 | GCA_003201555.1 | 70 | 15.45 |
| *Sulfitobacter* sp. EE36 | GCA_000152605.1 | 65 | 18.31 |
| *Sulfitobacter* sp. NAS-14.1 | GCA_000152645.1 | 70 | 17.46 |
| *Sulfitobacter pontiacus* EhN02 | GCA_001650835.1 | 60 | 17.29 |
| A_SAG1 | GCA_012395585.1 | 71 | 22.33 |
| A_SAG2 | GCA_012395565.1 | 64 | 21.99 |
| A_SAG3 | GCA_012395525.1 | 94 | 22.93 |
| A_SAG4 | GCA_012395545.1 | 75 | 22.19 |
| A_SAG5 | GCA_012395495.1 | 41 | 23.84 |
| A_SAG6 | GCA_012395485.1 | 76 | 24.13 |
| A_SAG7 | GCA_012395435.1 | 55 | 21.74 |
| A_SAG8 | GCA_012395455.1 | 14 | 12.50 |
| *Alteromonas macleodii* str. | GCA_003545575.1 | 67 | 20.74 |
| *Alteromonas macleodii* AD006 | GCA_000808595.1 | 106 | 22.94 |
| *Alteromonas macleodii* AD037 | GCA_000808635.1 | 108 | 23.38 |
| *Alteromonas macleodii* ATCC 27126 | GCA_000172635.2 | 109 | 23.44 |
| *Alteromonas macleodii* CUKW | GCA_001750305.1 | 106 | 20.19 |
| *Alteromonas macleodii* D7 | GCA_001562235.1 | 77 | 16.81 |
| *Alteromonas macleodii* HOT1A3 | GCA_001578515.1 | 91 | 18.96 |
| *Alteromonas macleodii* KCC01 | GCA_001750295.1 | 106 | 20.23 |
| *Alteromonas macleodii* KCP01 | GCA_001750365.1 | 106 | 20.23 |
| *Alteromonas macleodii* KCPu01 | GCA_001750315.1 | 106 | 20.19 |
| *Alteromonas macleodii* str. Balearic Sea AD45 | GCA_000300175.1 | 108 | 23.13 |
| *Alteromonas macleodii* str. Black Sea 11 | GCA_000299995.1 | 106 | 23.66 |
| *Alteromonas macleodii* str. English Channel 673 | GCA_000299955.1 | 98 | 21.30 |
| *Alteromonas macleodii* Te101 | GCA_002849875.1 | 99 | 20.33 |
| *Alteromonas* sp. I10 | GCA_003201515.1 | 108 | 23.13 |
| *Alteromonas* sp. W12 | GCA_001924945.1 | 102 | 22.17 |

**Table S2.** Unique metabolic properties of the hadal *Roseobacter* SAGs based on annotation by Cluster of Orthologous Groups (COG) and Rapid Annotation using Subsystems Technology (RAST)*^a^*.

| COG category*^b^* | COG function ID | COG function | RAST function |
| --- | --- | --- | --- |
| C | COG2421 | Acetamidase/formamidase | Acetamidase (EC 3.5.1.4) |
| C | COG0427 | Acyl-CoA hydrolase | Acetyl-CoA hydrolase/transferase family protein |
| C | COG3794 | Plastocyanin | Copper binding protein, plastocyanin/azurin family |
| C | COG2010 | Cytochrome c, mono- and diheme variants | cytochrome C6 |
| C | COG1359 | Quinol monooxygenase YgiN | hypothetical protein |
| C | COG1012 | Acyl-CoA reductase or other NAD-dependent aldehyde dehydrogenase | Non-phosphorylating glyceraldehyde-3-phosphate dehydrogenase (NADP) (EC 1.2.1.9) |
| C | COG3005 | Tetraheme cytochrome c subunit of nitrate or TMAO reductase | Cytochrome c-type protein NapC |
| C | COG0680 | Ni,Fe-hydrogenase maturation factor | Hydrogenase maturation protease (EC 3.4.24.-) |
| C | COG2010 | Cytochrome c, mono- and diheme variants | Cytochrome c55X precursor NirC |
| CO | COG0785 | Cytochrome c biogenesis protein CcdA | Cytochrome c-type biogenesis protein CcdA (DsbD analog) |
| D | COG1196 | Chromosome segregation ATPase | COG0840: Methyl-accepting chemotaxis protein |
| D | COG4641 | Spore maturation protein CgeB | Ucharacterized protein, CGEB homolog |
| E | COG0683 | ABC-type branched-chain amino acid transport system, periplasmic component | ABC transporter, periplasmic protein |
| E | COG4608 | ABC-type oligopeptide transport system, ATPase component | Dipeptide transport ATP-binding protein DppD (TC 3.A.1.5.2) |
| E | COG1878 | Kynurenine formamidase | Metal-dependent hydrolase |
| E | COG0665 | Glycine/D-amino acid oxidase (deaminating) | putative monomeric sarcosine oxidase |
| E | COG1231 | Monoamine oxidase | Tryptophan 2-monooxygenase (EC 1.13.12.3) |
| E | COG0010 | Arginase family enzyme | Agmatinase (EC 3.5.3.11) |
| E | COG0031 | Cysteine synthase | Cysteine synthase (EC 2.5.1.47) |
| E | COG0683 | ABC-type branched-chain amino acid transport system, periplasmic component | High-affinity leucine-specific transport system, periplasmic binding protein LivK (TC 3.A.1.4.1) |
| E | COG0174 | Glutamine synthetase | Glutamine synthetase type I (EC 6.3.1.2) |
| E | COG2113 | ABC-type proline/glycine betaine transport system, periplasmic component | L-proline glycine betaine binding ABC transporter protein ProX (TC 3.A.1.12.1) |
| E | COG1177 | ABC-type spermidine/putrescine transport system, permease component II | Putrescine transport system permease protein potI |
| E | COG0687 | Spermidine/putrescine-binding periplasmic protein | ABC transporter, periplasmic spermidine putrescine-binding protein PotD (TC 3.A.1.11.1) |
| E | COG0069 | Glutamate synthase domain 2 | Glutamate synthase [NADPH] large chain (EC 1.4.1.13) |
| ER | COG1063 | Threonine dehydrogenase or related Zn-dependent dehydrogenase | Sorbitol dehydrogenase (EC 1.1.1.14) |
| ET | COG0834 | ABC-type amino acid transport/signal transduction system, periplasmic component/domain | FIG01023883: hypothetical protein |
| ET | COG0834 | ABC-type amino acid transport/signal transduction system, periplasmic component/domain | FIG01023917: hypothetical protein |
| F | COG3194 | Ureidoglycolate hydrolase (allantoin degradation) | hypothetical protein |
| F | COG1051 | ADP-ribose pyrophosphatase YjhB, NUDIX family | MutT/nudix family protein |
| F | COG0034 | Glutamine phosphoribosylpyrophosphate amidotransferase | Glutamine amidotransferase protein GlxB (EC 2.4.2.-) |
| G | COG3090 | TRAP-type C4-dicarboxylate transport system, small permease component | TRAP dicarboxylate transporter, DctQ subunit, unknown substrate 3 |
| G | COG2376 | Dihydroxyacetone kinase | Dihydroxyacetone kinase, ATP-dependent (EC 2.7.1.29) |
| G | COG1653 | ABC-type glycerol-3-phosphate transport system, periplasmic component | Inositol transport system sugar-binding protein |
| G | COG4130 | Predicted sugar epimerase, xylose isomerase-like family | Inosose isomerase (EC 5.3.99.-) |
| G | COG1486 | Alpha-galactosidase/6-phospho-beta-glucosidase, family 4 of glycosyl hydrolase | Alpha-galactosidase (EC 3.2.1.22) |
| G | COG4573 | Tagatose-1,6-bisphosphate aldolase non-catalytic subunit AgaZ/GatZ | Tagatose-6-phosphate kinase AgaZ (EC 2.7.1.144) |
| GEPR | COG0477 | MFS family permease | transporter, putative |
| GER | COG0697 | Permease of the drug/metabolite transporter (DMT) superfamily | Permease of the drug/metabolite transporter (DMT) superfamily |
| H | COG0156 | 7-keto-8-aminopelargonate synthetase or related enzyme | 8-amino-7-oxononanoate synthase (EC 2.3.1.47) |
| H | COG1865 | Adenosylcobinamide amidohydrolase | Adenosylcobinamide amidohydrolase (EC 3.5.1.90) |
| H | COG2227 | 2-polyprenyl-3-methyl-5-hydroxy-6-metoxy-1,4-benzoquinol methylase | FIG01025284: hypothetical protein |
| H | COG2226 | Ubiquinone/menaquinone biosynthesis C-methylase UbiE | Methyltransferase |
| H | COG0043 | 3-polyprenyl-4-hydroxybenzoate decarboxylase | 3-polyprenyl-4-hydroxybenzoate carboxy-lyase (EC 4.1.1.-) |
| H | COG0163 | 3-polyprenyl-4-hydroxybenzoate decarboxylase | 3-polyprenyl-4-hydroxybenzoate carboxy-lyase UbiX (EC 4.1.1.-) |
| H | COG2226 | Ubiquinone/menaquinone biosynthesis C-methylase UbiE | SAM-dependent methyltransferase SCO3452 (UbiE paralog) |
| HR | COG2141 | Flavin-dependent oxidoreductase, luciferase family (includes alkanesulfonate monooxygenase SsuD and methylene tetrahydromethanopterin reductase) | Nitrilotriacetate monooxygenase component A (EC 1.14.13.-) |
| I | COG1804 | Crotonobetainyl-CoA:carnitine CoA-transferase CaiB and related acyl-CoA transferases | Dimethylsulfoniopropionate (DMSP) acyl CoA transferase DddD |
| I | COG2030 | Acyl dehydratase | hypothetical protein |
| I | COG3154 | Predicted lipid carrier protein YhbT, SCP2 domain | Sterol binding protein |
| I | COG2084 | 3-hydroxyisobutyrate dehydrogenase or related beta-hydroxyacid dehydrogenase | 3-hydroxyisobutyrate dehydrogenase (EC 1.1.1.31) (HIBADH) |
| I | COG1257 | Hydroxymethylglutaryl-CoA reductase | Hydroxymethylglutaryl-CoA reductase (EC 1.1.1.34) |
| I | COG1398 | Fatty-acid desaturase | Fatty acid desaturase (EC 1.14.19.1); Delta-9 fatty acid desaturase (EC 1.14.19.1) |
| I | COG0183 | Acetyl-CoA acetyltransferase | Acetyl-CoA acetyltransferase (EC 2.3.1.9) |
| I | COG1260 | Myo-inositol-1-phosphate synthase | Inositol-1-phosphate synthase (EC 5.5.1.4) |
| I | COG1960 | Acyl-CoA dehydrogenase related to the alkylation response protein AidB | Butyryl-CoA dehydrogenase (EC 1.3.99.2) |
| I | COG0671 | Membrane-associated phospholipid phosphatase | hypothetical membrane protein |
| I | COG0558 | Phosphatidylglycerophosphate synthase | CDP-diacylglycerol--glycerol-3-phosphate 3-phosphatidyltransferase (EC 2.7.8.5) |
| IQR | COG1028 | NAD(P)-dependent dehydrogenase, short-chain alcohol dehydrogenase family | 2,3-dihydroxy-2,3-dihydro-phenylpropionate dehydrogenase (EC 1.3.1.-) |
| J | COG0456 | Ribosomal protein S18 acetylase RimI and related acetyltransferases | acetyltransferase, GNAT family |
| J | COG0456 | Ribosomal protein S18 acetylase RimI and related acetyltransferases | hypothetical protein |
| J | COG0223 | Methionyl-tRNA formyltransferase | Methionyl-tRNA formyltransferase (EC 2.1.2.9) |
| J | COG2813 | 16S rRNA G1207 methylase RsmC | O-methyltransferase, family 2 |
| J | COG1234 | Ribonuclease BN, tRNA processing enzyme | Ribonuclease Z (EC 3.1.26.11) |
| J | COG3642 | tRNA A-37 threonylcarbamoyl transferase component Bud32 | Serine/threonine protein kinase |
| J | COG2110 | O-acetyl-ADP-ribose deacetylase (regulator of RNase III), contains Macro domain |  |
| J | COG1514 | 2'-5' RNA ligase | 2'-5' RNA ligase |
| J | COG0486 | tRNA U34 5-carboxymethylaminomethyl modifying GTPase MnmE/TrmE | hypothetical protein |
| JR | COG1418 | HD superfamily phosphodieaserase, includes HD domain of RNase Y | metal-dependent phosphohydrolase |
| K | COG1396 | Transcriptional regulator, contains XRE-family HTH domain | DNA-binding protein |
| K | COG2345 | Predicted transcriptional regulator, ArsR family | FIG00875025: hypothetical protein |
| K | COG2214 | Curved DNA-binding protein CbpA, contains a DnaJ-like domain | FIG033376: Heat shock protein DnaJ-like |
| K | COG1522 | DNA-binding transcriptional regulator, Lrp family | Heme d1 biosynthesis protein NirD / Heme d1 biosynthesis protein NirL |
| K | COG1522 | DNA-binding transcriptional regulator, Lrp family | Heme d1 biosynthesis protein NirG |
| K | COG1522 | DNA-binding transcriptional regulator, Lrp family | Heme d1 biosynthesis protein NirH |
| K | COG1510 | DNA-binding transcriptional regulator GbsR, MarR family | hypothetical protein |
| K | COG3609 | Transcriptional regulator, contains Arc/MetJ-type RHH (ribbon-helix-helix) DNA-binding domain | Miscellaneous; Unknown |
| K | COG2865 | Predicted transcriptional regulator, contains HTH domain | Predicted transcriptional regulator containing an HTH domain and an uncharacterized domain shared with the mammalian protein Schlafen |
| K | COG1510 | DNA-binding transcriptional regulator GbsR, MarR family | Probable transcriptional regulator |
| K | COG2207 | AraC-type DNA-binding domain and AraC-containing proteins | Transcriptional regulator, AraC family |
| K | COG1309 | DNA-binding transcriptional regulator, AcrR family | Transcriptional regulator, TetR family |
| K | COG1309 | DNA-binding transcriptional regulator, AcrR family | Transcriptional regulator, TetR family |
| K | COG5662 | Transmembrane transcriptional regulator (anti-sigma factor RsiW) | Transmembrane regulator protein PrtR |
| KG | COG1940 | Sugar kinase of the NBD/HSP70 family, may contain an N-terminal HTH domain | ROK family Glucokinase with ambiguous substrate specificity |
| KG | COG1349 | DNA-binding transcriptional regulator of sugar metabolism, DeoR/GlpR family | Transcriptional regulator of rhamnose utilization, DeoR family |
| KL | COG0553 | Superfamily II DNA or RNA helicase, SNF2 family | DEAD/DEAH box helicase-like protein |
| KL | COG0553 | Superfamily II DNA or RNA helicase, SNF2 family | Helicase |
| L | COG0587 | DNA polymerase III, alpha subunit | DNA polymerase III alpha subunit (EC 2.7.7.7) |
| L | COG1961 | Site-specific DNA recombinase related to the DNA invertase Pin | elements of external origin; phage-related functions and prophages |
| L | COG3593 | Predicted ATP-dependent endonuclease of the OLD family, contains P-loop ATPase and TOPRIM domains | FIG131328: Predicted ATP-dependent endonuclease of the OLD family |
| L | COG1961 | Site-specific DNA recombinase related to the DNA invertase Pin | hypothetical protein |
| L | COG3598 | RecA-family ATPase | RecA-family ATPase |
| L | COG1273 | Non-homologous end joining protein Ku, dsDNA break repair | Ku domain protein |
| LX | COG0582 | Integrase | hypothetical protein |
| LX | COG0582 | Integrase | Site-specific recombinase, phage integrase family |
| M | COG0438 | Glycosyltransferase involved in cell wall bisynthesis | Alpha-D-GlcNAc alpha-1,2-L-rhamnosyltransferase (EC 2.4.1.-) |
| M | COG0438 | Glycosyltransferase involved in cell wall bisynthesis | Glycosyl transferase, group 2 family protein |
| M | COG2327 | Polysaccharide pyruvyl transferase family protein WcaK | hypothetical protein |
| M | COG0668 | Small-conductance mechanosensitive channel | Mechanosensitive ion channel family protein |
| M | COG0451 | Nucleoside-diphosphate-sugar epimerase | Nucleoside-diphosphate-sugar epimerases |
| M | COG2834 | Outer membrane lipoprotein-sorting protein | Outer membrane lipoprotein-sorting protein |
| M | COG3980 | Spore coat polysaccharide biosynthesis protein SpsG, predicted glycosyltransferase | Pseudaminic acid cytidylyltransferase (EC 2.7.7.43) |
| M | COG3131 | Periplasmic glucans biosynthesis protein |  |
| M | COG0381 | UDP-N-acetylglucosamine 2-epimerase | UDP-N-acetylglucosamine 2-epimerase (EC 5.1.3.14) |
| M | COG4952 | L-rhamnose isomerase | L-rhamnose isomerase (EC 5.3.1.14) |
| M | COG1463 | ABC-type transporter Mla maintaining outer membrane lipid asymmetry, periplasmic component MlaD | ABC-type transport system involved in resistance to organic solvents, periplasmic component USSDB6C |
| M | COG2222 | Fructoselysine-6-P-deglycase FrlB and related proteins with duplicated sugar isomerase (SIS) domain | Galactosamine-6-phosphate isomerase (EC 5.3.1.-) |
| M | COG3659 | Carbohydrate-selective porin OprB | hypothetical protein |
| M | COG2173 | D-alanyl-D-alanine dipeptidase | D-alanyl-D-alanine dipeptidase |
| MDT | COG0464 | AAA+-type ATPase, SpoVK/Ycf46/Vps4 family | FIG00919194: hypothetical protein |
| MI | COG1887 | CDP-glycerol glycerophosphotransferase, TagB/SpsB family | putative integral membrane protein. |
| MR | COG3049 | Penicillin V acylase or related amidase, Ntn superfamily | Choloylglycine hydrolase (EC 3.5.1.24) |
| N | COG1360 | Flagellar motor protein MotB | Chemotaxis protein MotB-related protein |
| NW | COG3170 | Tfp pilus assembly protein FimV | RidA/YER057c/UK114 superfamily, group 3 |
| O | COG4232 | Thiol:disulfide interchange protein | FIG01024482: hypothetical protein |
| O | COG0826 | Collagenase-like protease, PrtC family | FIG139928: Putative protease |
| O | COG3118 | Negative regulator of GroEL, contains thioredoxin-like and TPR-like domains | hypothetical protein |
| O | COG0298 | Hydrogenase maturation factor | [NiFe] hydrogenase metallocenter assembly protein HypC |
| O | COG0409 | Hydrogenase maturation factor | [NiFe] hydrogenase metallocenter assembly protein HypD |
| O | COG0309 | Hydrogenase maturation factor | [NiFe] hydrogenase metallocenter assembly protein HypE |
| O | COG0068 | Hydrogenase maturation factor HypF (carbamoyltransferase) | [NiFe] hydrogenase metallocenter assembly protein HypF |
| O | COG2170 | Gamma-glutamyl:cysteine ligase YbdK, ATP-grasp superfamily | Carboxylate-amine ligase bll3764 (EC 6.3.-.-) |
| O | COG0826 | Collagenase-like protease, PrtC family | Collagenase and related proteases |
| O | COG0826 | Collagenase-like protease, PrtC family | peptidase, U32 family large subunit [C1] |
| P | COG4097 | Predicted ferric reductase | putative oxidoreductase |
| P | COG4771 | Outer membrane receptor for ferrienterochelin and colicins | TonB-dependent receptor |
| P | COG0053 | Divalent metal cation (Fe/Co/Zn/Cd) transporter | - |
| P | COG4771 | Outer membrane receptor for ferrienterochelin and colicins | Outer membrane receptor proteins, mostly Fe transport |
| P | COG0614 | ABC-type Fe3+-hydroxamate transport system, periplasmic component | Putative periplasmic substrate-binding transport protein |
| P | COG0715 | ABC-type nitrate/sulfonate/bicarbonate transport system, periplasmic component | ABC-type nitrate/sulfonate/bicarbonate transport systems, periplasmic components |
| P | COG4615 | ABC-type siderophore export system, fused ATPase and permease components | ABC transporter, ATP-binding protein |
| P | COG3142 | Copper homeostasis protein CutC | Cytoplasmic copper homeostasis protein cutC |
| P | COG4521 | ABC-type taurine transport system, periplasmic component | Taurine-binding periplasmic protein TauA |
| P | COG1814 | Predicted Fe2+/Mn2+ transporter, VIT1/CCC1 family | nodulin 21-related protein |
| Q | COG2931 | Ca2+-binding protein, RTX toxin-related | Alkaline phosphatase (EC 3.1.3.1) |
| Q | COG2931 | Ca2+-binding protein, RTX toxin-related | Alkaline phosphatase (EC 3.1.3.1) |
| Q | COG0346 | Catechol 2,3-dioxygenase or other lactoylglutathione lyase family enzyme | glyoxalase/bleomycin resistance protein/dioxygenase |
| Q | COG0346 | Catechol 2,3-dioxygenase or other lactoylglutathione lyase family enzyme | Glyoxalase/bleomycin resistance protein/dioxygenase precursor |
| Q | COG0346 | Catechol 2,3-dioxygenase or other lactoylglutathione lyase family enzyme | Lactoylglutathione lyase and related lyases |
| Q | COG2050 | Acyl-coenzyme A thioesterase PaaI, contains HGG motif | Phenylacetic acid degradation protein PaaD, thioesterase |
| Q | COG1228 | Imidazolonepropionase or related amidohydrolase | Prolidase (EC 3.4.13.9) |
| Q | COG4665 | TRAP-type mannitol/chloroaromatic compound transport system, small permease component | TRAP-type C4-dicarboxylate transport system, small permease component |
| Q | COG5285 | Ectoine hydroxylase-related dioxygenase, phytanoyl-CoA dioxygenase (PhyH) family | Protein involved in biosynthesis of mitomycin antibiotics/polyketide fumonisin |
| Q | COG3319 | Thioesterase domain of type I polyketide synthase or non-ribosomal peptide synthetase | Siderophore biosynthesis non-ribosomal peptide synthetase modules @ Bacillibactin synthetase component F (EC 2.7.7.-) |
| Q | COG1647 | Esterase/lipase | putative esterase/lipase |
| T | COG3300 | MHYT domain, NO-binding membrane sensor | carbon monoxide dehydrogenase operon C protein |
| T | COG2365 | Protein tyrosine/serine phosphatase | hypothetical protein |
| T | COG1073 | Fermentation-respiration switch protein FrsA, has esterase activity, DUF1100 family | hypothetical protein |
| T | COG2114 | Adenylate cyclase, class 3 | hypothetical protein |
| T | COG0589 | Nucleotide-binding universal stress protein, UspA family | Universal stress protein UspA and related nucleotide-binding proteins |
| T | COG2114 | Adenylate cyclase, class 3 | Adenylate cyclase (EC 4.6.1.1) |
| T | COG3476 | Tryptophan-rich sensory protein (mitochondrial benzodiazepine receptor homolog) | Regulatory protein TspO |
| T | COG3550 | Serine/threonine protein kinase HipA, toxin component of the HipAB toxin-antitoxin module | HipA protein |
| TE | COG0347 | Nitrogen regulatory protein PII | FIG01023534: hypothetical protein |
| TK | COG3707 | Two-component response regulator, AmiR/NasT family, consists of REC and RNA-binding antiterminator (ANTAR) domains | DNA-binding heavy metal response regulator |
| TK | COG2197 | DNA-binding response regulator, NarL/FixJ family, contains REC and HTH domains | hypothetical protein |
| U | COG3843 | Type IV secretory pathway, VirD2 components (relaxase) | hypothetical protein |
| UW | COG3847 | Flp pilus assembly protein, pilin Flp | Flp pilus assembly protein, pilin Flp |
| V | COG1566 | Multidrug resistance efflux pump | FIG01073238: hypothetical protein |
| V | COG3587 | Restriction endonuclease | Type III restriction enzyme, res subunit:DEAD/DEAH box helicase, N-terminal |
| V | COG4096 | Type I site-specific restriction endonuclease, part of a restriction-modification system | Type III restriction-modification enzyme, helicase subunit |
| V | COG1203 | CRISPR/Cas system-associated endonuclease/helicase Cas3 | CRISPR-associated helicase Cas3 |
| X | COG5614 | Bacteriophage head-tail adaptor | Bacteriophage head-tail adaptor |
| X | COG2826 | Transposase and inactivated derivatives, IS30 family | hypothetical protein |
| X | COG4220 | Phage DNA packaging protein, Nu1 subunit of terminase | hypothetical protein |
| X | COG5527 | Protein involved in initiation of plasmid replication | initiator RepB protein |
| X | COG3668 | Plasmid stabilization system protein ParE | Miscellaneous; Unknown |
| X | COG2801 | Transposase InsO and inactivated derivatives | Mobile element protein |
| X | COG3385 | IS4 transposase | Mobile element protein |
| X | COG2801 | Transposase InsO and inactivated derivatives | Mobile element protein |
| X | COG3747 | Phage terminase, small subunit | Phage terminase small subunit |
| X | COG3600 | Uncharacterized phage-associated protein |  |
| X | COG1783 | Phage terminase large subunit | Phage terminase, large subunit |
| X | COG3378 | Phage- or plasmid-associated DNA primase |  |
| X | COG1943 | REP element-mobilizing transposase RayT | Transposase and inactivated derivatives |
| R | COG1661 | Predicted DNA-binding protein with PD1-like DNA-binding motif | Bll4710 protein |
| R | COG2374 | Predicted extracellular nuclease | Endonuclease/exonuclease/phosphatase family protein |
| R | COG1917 | Cupin domain protein related to quercetin dioxygenase | FIG01023233: hypothetical protein |
| R | COG1917 | Cupin domain protein related to quercetin dioxygenase | Glutamate synthase [NADPH] large chain (EC 1.4.1.13) |
| R | COG2249 | Putative NADPH-quinone reductase (modulator of drug activity B) | Glutathione-regulated potassium-efflux system ancillary protein KefG |
| R | COG0535 | Radical SAM superfamily enzyme, MoaA/NifB/PqqE/SkfB family | Heme d1 biosynthesis protein NirJ |
| R | COG1672 | Predicted ATPase, archaeal AAA+ ATPase superfamily | hypothetical protein |
| R | COG1765 | Uncharacterized OsmC-related protein | hypothetical protein |
| R | COG4782 | Esterase/lipase superfamily enzyme | Lipoprotein, putative |
| R | COG3391 | DNA-binding beta-propeller fold protein YncE | Nitrite reductase associated c-type cytochorome NirN |
| R | COG0673 | Predicted dehydrogenase | Oxidoreductase, N-terminal |
| R | COG4889 | Predicted helicase | putative helicase |
| R | COG0628 | Predicted PurR-regulated permease PerM | Putative membrane protein |
| R | COG2522 | Predicted transcriptional regulator | Replication protein |
| R | COG0673 | Predicted dehydrogenase | Small Molecule Metabolism |
| R | COG0300 | Short-chain dehydrogenase | short-chain dehydrogenase/reductase SDR |
| R | COG2764 | Uncharacterized conserved protein PhnB, glyoxalase superfamily | PhnB protein; putative DNA binding 3-demethylubiquinone-9 3-methyltransferase domain protein |
| R | COG3324 | Predicted enzyme related to lactoylglutathione lyase | glyoxalase family protein |
| R | COG3324 | Predicted enzyme related to lactoylglutathione lyase | Glyoxalase family protein |
| R | COG0400 | Predicted esterase | Carboxylesterase (EC 3.1.1.1) |
| R | COG1033 | Predicted exporter protein, RND superfamily | Predicted exporter of the RND superfamily |
| R | COG3571 | Predicted hydrolase of the alpha/beta-hydrolase fold | Esterase/lipase/thioesterase family active site |
| R | COG3391 | DNA-binding beta-propeller fold protein YncE | Cytochrome cd1 nitrite reductase (EC:1.7.2.1) |
| R | COG3218 | ABC-type uncharacterized transport system, auxiliary component | Membrane lipoprotein lipid attachment site containing protein USSDB6D |
| R | COG3391 | DNA-binding beta-propeller fold protein YncE | Heme d1 biosynthesis protein NirF |
| S | COG5293 | Uncharacterized protein YydD, contains DUF2326 domain | ATPase involved in DNA repair |
| S | COG0730 | Uncharacterized membrane protein YfcA | Bll7429 protein |
| S | COG4991 | Uncharacterized conserved protein YraI | Conserved domain protein |
| S | COG5469 | Predicted metal-binding protein | FIG00777107: hypothetical protein |
| S | COG3597 | Uncharacterized conserved protein, DUF697 family | hypothetical protein |
| S | COG4898 | Uncharacterized protein | hypothetical protein |
| S | COG1511 | Uncharacterized membrane protein YhgE, phage infection protein (PIP) family | Phage tail length tape-measure protein 1 |
| S | COG3832 | Uncharacterized conserved protein YndB, AHSA1/START domain | Probable glutathione S-transferase-related transmembrane protein (EC 2.5.1.18) |
| S | COG3366 | Uncharacterized protein | putative membrane protein |
| S | COG3603 | Uncharacterized protein | hypothetical protein |
| S | COG3242 | Uncharacterized conserved protein YjeT, DUF2065 family | Putative inner membrane protein YjeT (clustered with HflC) |
| S | COG3310 | Uncharacterized protein | hypothetical protein |

*^a^*Only unique gene clusters (GCs) containing >4 orthologous genes are listed.

*^b^*COG categories are represented by letters. C, energy production and conversion; D, cell cycle control, cell division, chromosome partitioning; E, amino acid transport and metabolism; F, nucleotide transport and metabolism; G, carbohydrate transport and metabolism; H, coenzyme transport and metabolism; I, lipid transport and metabolism; J, translation, ribosomal structure and biogenesis; K, transcription; L, replication, recombination and repair; M, cell wall/membrane/envelope biogenesis; N, cell motility; O, posttranslational modification, protein turnover, chaperones; P, inorganic ion transport and metabolism; Q, secondary metabolites biosynthesis, transport and catabolism; R, general function prediction only; S, function unknown; T, signal transduction mechanisms; U, intracellular trafficking, secretion, and vesicular transport; V, defense mechanisms; W, extracellular structures; X, mobilome: prophages, transposons. Multiple COG categories are listed for GCs that can be assigned to multiple functional categories.

**Table S3.** Unique metabolic properties of the hadal *Alteromonas* SAGs based on annotation by Cluster of Orthologous Groups (COG) and Rapid Annotation using Subsystems Technology (RAST)*^a^*.

| COG category*^b^* | COG function ID | COG function | RAST function |
| --- | --- | --- | --- |
| CP | COG5013 | Nitrate reductase alpha subunit | Respiratory nitrate reductase alpha chain (EC 1.7.99.4) |
| CP | COG1140 | Nitrate reductase beta subunit | Respiratory nitrate reductase beta chain (EC 1.7.99.4) |
| CP | COG2181 | Nitrate reductase gamma subunit | Respiratory nitrate reductase gamma chain (EC 1.7.99.4) |
| CPO | COG2180 | Nitrate reductase assembly protein NarJ, required for insertion of molybdenum cofactor | Respiratory nitrate reductase delta chain (EC 1.7.99.4) |
| D | COG4641 | Spore maturation protein CgeB | Glycosyl transferase, family 2 |
| D | COG1196 | Chromosome segregation ATPase | hypothetical protein |
| D | COG1196 | Chromosome segregation ATPase | Purine NTPase |
| E | COG2755 | Lysophospholipase L1 or related esterase | Sialic acid-specific 9-O-acetylesterase |
| EH | COG0028 | Acetolactate synthase large subunit or other thiamine pyrophosphate-requiring enzyme | Pyruvate decarboxylase (EC 4.1.1.1); Indole-3-pyruvate decarboxylase (EC 4.1.1.74) |
| EM | COG0329 | Dihydrodipicolinate synthase/N-acetylneuraminate lyase | N-acetylneuraminate lyase (EC 4.1.3.3) |
| F | COG1328 | Anaerobic ribonucleoside-triphosphate reductase | Ribonucleotide reductase of class III (anaerobic), large subunit (EC 1.17.4.2) |
| G | COG3347 | Rhamnose utilisation protein RhaD, predicted bifunctional aldolase and dehydrogenase | Predicted rhamnulose-1-phosphate aldolase (EC 4.1.2.19) / Predicted lactaldehyde dehydrogenase (EC 1.2.1.22) |
| G | COG1070 | Sugar (pentulose or hexulose) kinase | Rhamnulokinase RhaK in alpha-proteobacteria (EC 2.7.1.5) |
| G | COG3010 | Putative N-acetylmannosamine-6-phosphate epimerase | N-acetylmannosamine-6-phosphate 2-epimerase (EC 5.1.3.9) |
| G | COG0363 | 6-phosphogluconolactonase /Glucosamine-6-phosphate isomerase/deaminase | Glucosamine-6-phosphate deaminase (EC 3.5.99.6) |
| G | COG4225 | Rhamnogalacturonyl hydrolase YesR | Rhamnogalacturonides degradation protein RhiN |
| G | COG2211 | Na+/melibiose symporter or related transporter | Rhamnogalacturonide transporter RhiT |
| I | COG2267 | Lysophospholipase, alpha-beta hydrolase superfamily | Alpha/beta hydrolase fold |
| I | COG0657 | Acetyl esterase/lipase | putative lipase/esterase |
| I | COG3154 | Predicted lipid carrier protein YhbT, SCP2 domain | sterol binding protein |
| IQ | COG0318 | Acyl-CoA synthetase (AMP-forming)/AMP-acid ligase II | Long-chain-fatty-acid--CoA ligase (EC 6.2.1.3) |
| K | COG1396 | Transcriptional regulator, contains XRE-family HTH domain | Transcriptional regulator, XRE family |
| KG | COG1940 | Sugar kinase of the NBD/HSP70 family, may contain an N-terminal HTH domain | N-acetylmannosamine kinase (EC 2.7.1.60) |
| KL | COG0553 | Superfamily II DNA or RNA helicase, SNF2 family | DEAD/DEAH box helicase-like protein |
| KL | COG1061 | Superfamily II DNA or RNA helicase | FOG: GGDEF domain |
| L | COG2356 | Endonuclease I | Endonuclease I precursor (EC 3.1.21.1) |
| LX | COG0582 | Integrase | Integrase |
| LX | COG0582 | Integrase | Phage integrase |
| M | COG3055 | N-acetylneuraminic acid mutarotase | Predicted sialic acid transporter |
| M | COG4952 | L-rhamnose isomerase | L-rhamnose isomerase (EC 5.3.1.14) |
| M | COG1136 | ABC-type lipoprotein export system, ATPase component | ABC transporter ATP-binding protein YvcR |
| M | COG3254 | L-rhamnose mutarotase | L-rhamnose mutarotase |
| O | COG0826 | Collagenase-like protease, PrtC family | FIG139928: Putative protease |
| O | COG1858 | Cytochrome c peroxidase | Probable cytochrome-c peroxidase (EC 1.11.1.5) |
| O | COG0760 | Parvulin-like peptidyl-prolyl isomerase | Peptidyl-prolyl cis-trans isomerase PpiD (EC 5.2.1.8) |
| O | COG1180 | Pyruvate-formate lyase-activating enzyme | Ribonucleotide reductase of class III (anaerobic), activating protein (EC 1.97.1.4) |
| O | COG2846 | Iron-sulfur cluster repair protein YtfE, RIC family, contains ScdAN and hemerythrin domains | Nitric oxide-dependent regulator DnrN or NorA |
| P | COG4771 | Outer membrane receptor for ferrienterochelin and colicins | TonB-dependent receptor |
| P | COG4148 | ABC-type molybdate transport system, ATPase component | Molybdenum transport ATP-binding protein ModC (TC 3.A.1.8.1) |
| P | COG0725 | ABC-type molybdate transport system, periplasmic component | Molybdenum ABC transporter, periplasmic molybdenum-binding protein ModA (TC 3.A.1.8.1) |
| P | COG2223 | Nitrate/nitrite transporter NarK | Nitrate/nitrite transporter |
| P | COG2223 | Nitrate/nitrite transporter NarK | Nitrate/nitrite transporter |
| P | COG2223 | Nitrate/nitrite transporter NarK | Nitrate/nitrite transporter |
| P | COG0753 | Catalase | Catalase (EC 1.11.1.6) |
| P | COG1230 | Co/Zn/Cd efflux system component | Cobalt-zinc-cadmium resistance protein CzcD |
| T | COG0517 | CBS domain | CBS domain pair, putative |
| T | COG4585 | Signal transduction histidine kinase | hypothetical protein |
| T | COG3850 | Signal transduction histidine kinase, nitrate/nitrite-specific | Nitrate/nitrite sensor protein (EC 2.7.3.-) |
| TK | COG3706 | Two-component response regulator, PleD family, consists of two REC domains and a diguanylate cyclase (GGDEF) domain | diguanylate cyclase/phosphodiesterase (GGDEF & EAL domains) with PAS/PAC sensor(s) |
| U | COG0823 | Periplasmic component of the Tol biopolymer transport system | Oligogalacturonate lyase (EC 4.2.2.6) |
| V | COG4823 | Abortive infection bacteriophage resistance protein | Abortive infection bacteriophage resistance protein |
| V | COG2161 | Antitoxin component YafN of the YafNO toxin-antitoxin module, PHD/YefM family | Prevent-host-death family protein |
| V | COG3587 | Restriction endonuclease | Type III restriction enzyme, res subunit:DEAD/DEAH box helicase, N-terminal |
| V | COG3213 | Uncharacterized protein involved in response to NO | NnrS protein involved in response to NO |
| X | COG2826 | Transposase and inactivated derivatives, IS30 family | Mobile element protein |
| X | COG1943 | REP element-mobilizing transposase RayT | Mobile element protein |
| X | COG3668 | Plasmid stabilization system protein ParE | YafQ toxin protein |
| X | COG3039 | Transposase and inactivated derivatives, IS5 family | Mobile element protein |
| X | COG3039 | Transposase and inactivated derivatives, IS5 family | Mobile element protein |
| S | COG3094 | Uncharacterized membrane protein SirB2 | FIG002082: Protein SirB2 |
| S | COG4688 | Uncharacterized protein | hypothetical protein |
| S | COG2461 | Uncharacterized conserved protein, DUF438 domain, may contain hemerythrin domain | putative protein |

*^a^*Only unique gene clusters (GCs) containing >4 orthologous genes are listed.

*^b^*COG categories are represented by letters. C, energy production and conversion; D, cell cycle control, cell division, chromosome partitioning; E, amino acid transport and metabolism; F, nucleotide transport and metabolism; G, carbohydrate transport and metabolism; H, coenzyme transport and metabolism; I, lipid transport and metabolism; K, transcription; L, replication, recombination and repair; M, cell wall/membrane/envelope biogenesis; O, posttranslational modification, protein turnover, chaperones; P, inorganic ion transport and metabolism; S, function unknown; T, signal transduction mechanisms; U, intracellular trafficking, secretion, and vesicular transport; V, defense mechanisms; X, mobilome: prophages, transposons. Multiple COG categories are listed for GCs that can be assigned to multiple functional categories.

**Table S4.** Summary of the number of plasmid replication related genes in each recovered SAG based on the RAST annotation.

|  | RepA | RepB | RepC | ParA | ParB | Uncertain proteins involved in the initiation of plasmid replication |
| --- | --- | --- | --- | --- | --- | --- |
| R_SAG1 | 4 | 3 | 1 | 1 | 1 | 0 |
| R_SAG2 | 5 | 3 | 1 | 1 | 2 | 0 |
| R_SAG3 | 2 | 3 | 2 | 0 | 0 | 0 |
| R_SAG4 | 1 | 2 | 2 | 1 | 0 | 2 |
| R_SAG5 | 1 | 0 | 0 | 3 | 1 | 0 |
| R_SAG6 | 1 | 1 | 1 | 1 | 0 | 0 |
| R_SAG7 | 4 | 3 | 1 | 1 | 1 | 1 |
| R_SAG8 | 2 | 2 | 0 | 0 | 0 | 0 |
| R_SAG9 | 0 | 0 | 0 | 1 | 1 | 0 |
| R_SAG10 | 0 | 0 | 0 | 0 | 0 | 0 |
| A_SAG1 | 0 | 0 | 0 | 3 | 0 | 0 |
| A_SAG2 | 0 | 0 | 0 | 2 | 0 | 0 |
| A_SAG3 | 0 | 0 | 0 | 4 | 1 | 0 |
| A_SAG4 | 0 | 0 | 0 | 2 | 0 | 0 |
| A_SAG5 | 0 | 0 | 0 | 2 | 1 | 0 |
| A_SAG6 | 0 | 0 | 0 | 2 | 0 | 0 |
| A_SAG7 | 0 | 0 | 0 | 3 | 0 | 0 |
| A_SAG8 | 0 | 0 | 0 | 1 | 0 | 0 |

RepABC, plasmid replication protein; ParAB, plasmid partitioning protein.
